# Systematic Evaluation of Whole Genome Sequence-Based Predictions of *Salmonella* Serotype and Antimicrobial Resistance

**DOI:** 10.3389/fmicb.2020.00549

**Published:** 2020-04-03

**Authors:** Ashley L. Cooper, Andrew J. Low, Adam G. Koziol, Matthew C. Thomas, Daniel Leclair, Sandeep Tamber, Alex Wong, Burton W. Blais, Catherine D. Carrillo

**Affiliations:** ^1^Research and Development, Ottawa Laboratory (Carling), Canadian Food Inspection Agency, Ottawa, ON, Canada; ^2^Department of Biology, Carleton University, Ottawa, ON, Canada; ^3^Microbial Contaminants, Canadian Food Inspection Agency, Calgary, AB, Canada; ^4^Ecotoxicology and Wildlife Health Division, Environment and Climate Change Canada, Ottawa, ON, Canada; ^5^Microbiology Research Division, Bureau of Microbial Hazards, Health Canada, Ottawa, ON, Canada

**Keywords:** *Salmonella*, antimicrobial resistance, serotyping, whole-genome sequence, *nuoF*, phenotype, genotype

## Abstract

Whole-genome sequencing (WGS) is used increasingly in public-health laboratories for typing and characterizing foodborne pathogens. To evaluate the performance of existing bioinformatic tools for *in silico* prediction of antimicrobial resistance (AMR) and serotypes of *Salmonella enterica*, WGS-based genotype predictions were compared with the results of traditional phenotyping assays. A total of 111 *S. enterica* isolates recovered from a Canadian baseline study on broiler chicken conducted in 2012-2013 were selected based on phenotypic resistance to 15 different antibiotics and isolates were subjected to WGS. Both SeqSero2 and SISTR accurately determined *S. enterica* serotypes, with full matches to laboratory results for 87.4 and 89.2% of isolates, respectively, and partial matches for the remaining isolates. Antimicrobial resistance genes (ARGs) were identified using several bioinformatics tools including the Comprehensive Antibiotic Resistance Database – Resistance Gene Identifier (CARD-RGI), Center for Genomic Epidemiology (CGE) ResFinder web tool, Short Read Sequence Typing for Bacterial Pathogens (SRST2 v 0.2.0), and *k*-mer alignment method (KMA v 1.17). All ARG identification tools had ≥ 99% accuracy for predicting resistance to all antibiotics tested except streptomycin (accuracy 94.6%). Evaluation of ARG detection in assembled versus raw-read WGS data found minimal observable differences that were gene- and coverage- dependent. Where initial phenotypic results indicated isolates were sensitive, yet ARGs were detected, repeat AMR testing corrected discrepancies. All tools failed to find resistance-determining genes for one gentamicin- and two streptomycin-resistant isolates. Further investigation found a single nucleotide polymorphism (SNP) in the *nuoF* coding region of one of the isolates which may be responsible for the observed streptomycin-resistant phenotype. Overall, WGS-based predictions of AMR and serotype were highly concordant with phenotype determination regardless of computational approach used.

## Introduction

The overuse of antibiotics in hospitals, the community, and agriculture is believed to have accelerated the emergence of multi-drug resistant microorganisms (WHO, 2017). This has resulted in increasing rates of antimicrobial resistance (AMR) globally posing a serious threat to public health. Without effective antibiotics to treat infectious diseases, healthcare costs, illness and mortality rates will rise. AMR surveillance programs provide data on the presence and emergence of AMR in the food production continuum (Dutil et al., 2010). In Canada, the Canadian Integrated Program for Antimicrobial Resistance Surveillance (CIPARS) monitors trends in antimicrobial use and resistance in selected bacterial organisms isolated from human, animal, and food sources across Canada (Government of Canada [PHAC], 2007). Isolated organisms are tested for antibiotic susceptibility using phenotypic tests to determine the minimum inhibitory concentrations (MICs) of antimicrobials that are significant to public health.

A variety of AMR mechanisms have been characterized, including production of proteins or enzymes that inactivate or modify the antimicrobial, alteration of the antimicrobial target, reduced uptake, increased efflux, and overproduction of the target (Blair et al., 2015; Chan, 2016). Some bacteria are intrinsically resistant to certain antimicrobials through functional or structural characteristics (e.g., absence of target) (Blair et al., 2015). Alternatively, AMR can be acquired or developed through spontaneous mutation, horizontal gene transfer, and genetic recombination, all of which can provide a competitive advantage (Laxminarayan et al., 2013; Blair et al., 2015; Chan, 2016). Recent studies have identified a large number of genes responsible for intrinsic and/or acquired AMR in microorganisms (van Hoek et al., 2011; Blair et al., 2015).

The increasing affordability of whole genome sequencing (WGS) has resulted in the feasibility of whole genome-bacterial sequencing in clinical and food testing laboratories. Prediction of bacterial phenotypes based on WGS is convenient, rapid, and has many beneficial applications including use in outbreak investigations, diagnostics, and epidemiological surveillance (Zankari, 2014; Knowles et al., 2016; Edirmanasinghe et al., 2017; Carrillo et al., 2019). This has led to the development of a number of bioinformatic tools for predicting bacterial phenotypes, including AMR profiles and serotype (Zankari et al., 2012, 2017; McArthur et al., 2013; Gupta et al., 2014; Inouye et al., 2014; Zhang et al., 2015, 2019; Yoshida et al., 2016). WGS analysis for AMR has the advantage of providing the full complement of resistance genes present in an isolate as well as the characterization of mutations that might confer resistance. Additional benefits include the ability to analyze a larger number of strains, as well as retrieve and re-analyze existing sequences, when new bioinformatics tools are developed and new genes are discovered, without time consuming culturing as is required for phenotypic testing.

There have been several investigations conducted to establish the concordance of AMR prediction based on detection of genetic markers and phenotypic resistance (Randall et al., 2004; Boerlin et al., 2005; Rosengren et al., 2009; Licker et al., 2015; Tyson et al., 2015, 2016; McDermott et al., 2016). WGS-based AMR prediction has been shown to be highly accurate for *Salmonella* and other organisms using custom AMR gene (ARG) databases (Tyson et al., 2015, 2016; McDermott et al., 2016; Zhao et al., 2016). Recognizing the need for common AMR prediction tools, a number of gene prediction databases are now available to the scientific community (Zankari et al., 2012; McArthur et al., 2013; Gupta et al., 2014; Inouye et al., 2014; Clausen et al., 2018; Feldgarden et al., 2019). However, studies including comprehensive comparison of more than two tools are limited (Gupta et al., 2014; Feldgarden et al., 2019; Doyle et al., 2020).

Although surveillance studies have shown an increase in overall *Salmonella* antimicrobial resistance over time (Su et al., 2004), the resistance rate varies between different *Salmonella* serotypes, with different antimicrobials, and with variations in phage presence (Zhao et al., 2007; Hong et al., 2016; Yoon et al., 2017). Whereas clinical isolates of *S. enterica* ser. Typhimurium and *S. enterica* ser. Heidelberg from 2004-2012 were found to have the highest levels of clinically important resistance (29.1 and 24.8%, respectively), analyses of veterinary *Salmonella* isolates in the United States from 2002 to 2003 found *S. enterica* ser. Uganda, *S. enterica* ser. Agona, and *S. enterica* ser. Newport commonly exhibited multidrug resistance (MDR) (Zhao et al., 2007). The correlation between AMR and certain *Salmonella* serotypes highlights the importance of monitoring and tracking in order to detect trends and inform policy for mitigating the impact of AMR.

*Salmonella* isolates are classified by serological reaction-based detection of somatic O antigens and phase variable flagellar H antigens H1 and H2 (Shipp and Rowe, 1980). The combination or formula of expressed antigens is then used to identify a serotype based on the White-Kauffmann-Le Minor scheme (Grimont and Weill, 2007). As serology-based serotyping is expensive, labor intensive, and time consuming, molecular methods and bead-based array assays have been developed (Muñoz et al., 2010; Bopp et al., 2016; Yoshida et al., 2016). Yet these techniques are still limited to identification of a portion of the approximately 2,500 *Salmonella* serotypes (Grimont and Weill, 2007; Zhang et al., 2015, 2019; Yoshida et al., 2016). WGS has the potential to allow rapid cost-effective identification of *Salmonella* isolates. The applications SeqSero and *Salmonella in silico* Typing Resource (SISTR) have recently been developed and evaluated for *in silico* determination of *Salmonella* serotypes using WGS data (Zhang et al., 2015, 2019; Yoshida et al., 2016; Yachison et al., 2017; Robertson et al., 2018; Uelze et al., 2020).

As the use of WGS-based analytical approaches for the characterization of bacterial pathogens to support public health investigations increases, it is critical to assess the reliability of tools developed for this purpose. This study provides a comparative analysis of the performance of publically available bioinformatics tools to accurately predict serotype and antimicrobial resistance of 111 *Salmonella* isolated in Canada using assembled genomes and raw sequence reads. The sequence coverage requirements for the accurate detection of AMR are also investigated.

## Materials and Methods

### Growth and Maintenance of *Salmonella* Strains

The *Salmonella* spp. isolates (*n* = 111) used in this study were selected from 2554 *Salmonella* strains collected between December 2012 and December 2013 by the Canadian Food Inspection Agency (CFIA) in collaboration with industry, federal and provincial partners as part of the national Microbiological Baseline Study (MBS) in Broiler Chickens (Government of Canada [CFIA], 2016). Isolates were recovered in accordance with the Food Safety and Inspection Service (FSIS) method MLG 4.05 as described in detail in the MBS report (Government of Canada [CFIA], 2016). *Salmonella* spp. isolates were submitted to the Public Health Agency of Canada (PHAC) – Laboratory of Foodborne Zoonoses in Guelph Ontario for serotyping and antimicrobial susceptibility testing. A total of 58 phenotypically resistant *S. enterica* and 53 phenotypically sensitive *S. enterica*, comprising 42 different serotypes, were selected for WGS based on differing resistance profiles (resistant to different antimicrobials in different combinations). Where possible an attempt was made to match a resistant strain with a sensitive strain of the same serotype. All strains were stored at −80°C in 15% glycerol and were plated on Brain-Heart Infusion agar (BHI) (Oxoid, Nepean, ON, Canada) and incubated overnight (14–16 h) at 37°C prior to use.

### Traditional Serotyping and Antimicrobial Susceptibility Testing

All strains used in this study were previously serotyped using traditional methods at the PHAC *Salmonella* Reference Laboratory (Guelph, ON, Canada). Standard methods were used to determine antigenic formula of each strain (Shipp and Rowe, 1980), and serotypes were assigned based on the White-Kauffmann-Le Minor scheme (Grimont and Weill, 2007).

Strains had also been previously tested for antimicrobial resistance by means of the broth microdilution method using the Sensititre Vizion^TM^ automated system (Trek Diagnostic Systems, Cleveland, OH, United States) at PHAC as described by the Canadian Integrated Program for Antimicrobial Resistance Surveillance (CIPARS) (Government of Canada, 2013). Briefly, the CMV2AGNF plate was used to test for resistance to 15 antimicrobials: gentamicin, GEN; kanamycin, KAN; streptomycin, STR; amoxicillin-clavulanic acid, AMC; cefoxitin, FOX; ceftiofur, TIO; ceftriaxone, CRO; ampicillin, AMP; chloramphenicol, CHL; sulfisoxazole, SOX; trimethoprim-sulfamethoxazole, SXT; tetracycline, TCY; nalidixic acid, NAL; and ciprofloxacin, CIP. Isolates were streaked on Mueller Hinton (MH) or MacConkey agar and incubated at 36°C for 18 to 24 h. One colony was selected from each plate, re-streaked for purification, and incubated; a 0.5-McFarland suspension was prepared by transferring growth from the agar plates to 5.0 mL of sterile, demineralized water. Ten microliters of suspension were transferred to 10 mL of MH broth (MHB) and dispensed onto CMV2AGNF testing plates at 50 μL per well and sealed. Plates were read automatically with the plate reading system after18 h incubation at 36°C. Breakpoints for resistance determination were determined according to CLSI guidelines M100-S23 and M31-A3 unless stated otherwise (Clinical and Laboratory Standards Institute, 2008, 2013).

### gDNA Isolation and Whole-Genome Sequencing (WGS)

For each isolate a single colony was transferred from BHI agar to 800 μL of BHI broth (Oxoid, Ottawa, ON, Canada) and incubated at 37°C for 3 h following which genomic DNA was isolated from 400 μL of broth culture using the Promega Maxwell^®^ 16 Cell DNA purification kit (Promega, Madison, WI, United States). Double-stranded genomic DNA was quantified using the Quant-iT^TM^ High Sensitivity Assay kit (Life Technologies Inc., Burlington, ON, Canada) according to the manufacturers’ recommendations. Sequencing libraries were constructed using the Nextera XT DNA sample preparation and the Nextera XT Index Kits (Illumina, Inc., San Diego, CA, United States) and paired-end sequencing was performed on the Illumina MiSeq platform, using 600-cycle MiSeq reagent kits (v3) with 5% PhiX control (Illumina Inc.).

### Bioinformatic Analysis

Raw sequencing read quality was assessed with FastQC version 0.11.8 (Andrews, 2010). Quality trimming was performed with BBDuk from BBTools version 38.22 (Bushnell, 2014) with the following parameters: trim quality of 10 and removal of reads below 50 bp long. Error correction was performed using tadpole version 8.22 (Bushnell, 2014) in ‘correct’ mode with default parameters. Sequences were checked for contamination using ConFindr 0.5.0 with default parameters (Low et al., 2019). Contigs were assembled from the trimmed and error-corrected reads using SKESA version 2.3.0 with the vector percent argument disabled (Souvorov et al., 2018). For assembled versus raw-read analyses where SPAdes assemblies were used, the same trimming and error correction steps were performed, and assemblies were created using SPAdes version 3.12.0 on default settings with the –only-assembler option (Bankevich et al., 2012). Pilon version 1.22 (Walker et al., 2014) was used to perform one round of automatic assembly improvement, and quality was assessed with Qualimap version 2.2.2 (García-Alcalde et al., 2012; Okonechnikov et al., 2016). A targeted minimum sequence coverage of 20X and minimum Phred quality score of 10 was used for sequence data. Plasmids were predicted and reconstructed from assembled genomes using the MOB-recon tool from MOB-suite v 1.4.1 (Robertson and Nash, 2018).

Serotyping of *Salmonella* spp. *in silico* was conducted using both raw reads and assembled genomes with SeqSero version 2 (SeqSero2), and with assemblies using SISTR developed by Zhang et al. (2015, 2019) and Yoshida et al. (2016), respectively. SISTR “overall” serovar predictions were used, as described by Yoshida et al. (2016). Analysis of separated paired end raw reads with SeqSero2 was conducted using both raw reads allele micro-assembly mode and k-mer mode, while assemblies were analyzed using the k-mer mode. Serotype predictions were compared to laboratory results and results were interpreted according to categories described by Yachison et al. (2017). Briefly, matches that were concordant with laboratory results were categorized as “full”. In cases where multiple serotypes were predicted (including the laboratory result), matches were categorized as “inconclusive”, and in cases where results differed because one or more of the antigen genes were not expressed and therefore not detected by laboratory methods, results were categorized as “incongruent”. Results were considered “incorrect” in cases where serovar predictions were different from the laboratory results.

Single nucleotide polymorphism (SNP) analysis of phenotypically resistant strains OLC2536, OLC2644, and OLC2626 with closely related sensitive strains was conducted using the Single Nucleotide Variant PHYLogenomics (SNVPhyl) pipeline version 1.0.1 (Petkau et al., 2017) with the reference set as the sensitive strain. High-quality SNPs had a minimum coverage of 5 reads, with 75% of reads supporting the SNP identification, and a filter density window of 500 with a density threshold of 2.

### ARG Identification in WGSs

Resistance genes were identified using each of the tools described in Table 1. The CARD-RGI tool was installed using bioconda from https://card.mcmaster.ca/download (Grüning et al., 2018). CGE’s PointFinder and ResFinder v2.1 web tools with default settings, threshold for%ID 90% and minimum length 60%, were used for analyses. The NCBI Antimicrobial Resistance Reference Gene Database (Bioproject PRJNA313047) (NCBI-AMR db) was downloaded from NCBI on May 29, 2018. The ARG-Annot and ResFinder databases for use with SRST2 (v 0.2.0) were downloaded from the SRST2 github^1^. The ResFinder database for use with KMA was installed via bitbucket with the KMA v1.0 tool as per author’s instructions. For all tools, ARGs were identified using a minimum cutoff of 90% nucleotide identity over a minimum length of 60% except for investigations of genotype-phenotype discrepancies where the select minimum length was lowered from 60% to 40%, and where stated otherwise.

**TABLE 1:** Characteristics of ARG detection tools.

Tool	Database^a^	Last Update prior this publication^b^	Supported Sequence Format	Originator	References
ResFinder v2.1 (web tool)		2019	Fasta	CGE	Zankari et al., 2012
KMA v1.17	ResFinder	2018	Fasta, Fastq	CGE	Clausen et al., 2018
	NCBI	2018			
SRST2	ResFinder	2014	Fastq	University of Melbourne	Gupta et al., 2014; Inouye et al., 2014
	ARG-Annot	2016-07			
	NCBI	2018			
CARD-RGI		2019	Fasta	McMaster University	McArthur et al., 2013

*Abbreviations: ARG, antimicrobial resistance gene; ResFinder, Resistance Finder; CGE, Center for Genomic Epidemiology; KMA, *k*-mer alignment method; SRST2, short read sequence typer v.0.2.0; ARG-Annot, antimicrobial resistance gene annotation; NCBI, National Center for Biotechnology Information Bacterial Antimicrobial Resistance Reference Gene Database; CARD-RGI, comprehensive Antibiotic Resistance Database Resistance Gene Identifier. ^*a*^ Database(s) used with tool (if multiple options were possible). ^*b*^ Year provided is for the database version used in this study.*

For chromosomal structural gene and SNP mutations CGE’s PointFinder program for identifying chromosomal mutations (now part of ResFinder) was used to investigate known mutations, while BLAST was used to investigate genes where SNPs were found in resistant strains compared to sensitive strains, and possibly conferred resistance (Camacho et al., 2009; Zankari et al., 2017). Sequences with < 100% amino acid identity to the DNA gyrase subunit A gene (*gyrA*) were reviewed to determine whether they matched known nalidixic acid-resistant (NalR) mutations (Table 2). This analysis was also conducted for the 111 *S. enterica* strains to search for mutations in *aroD, cyoB, cyoC, fusA, glnA, gidB, ispA, nuoE, nuoF, prfB, rpsL, trkH, ubiA, ubiE*, and *ubiF* genes which have a reported role in conversion of *Salmonella* to a small colony variant (SCV) phenotype and/or confer streptomycin resistance (Soballe and Poole, 1999; Springer et al., 2001; Cano et al., 2003; Okamoto et al., 2007; Koskiniemi et al., 2011; Lázár et al., 2013; Li et al., 2016; Aurass et al., 2017).

**TABLE 2:** Identification of single nucleotide variations resulting in non-synonymous mutations.

Gene	Isolate	Nucleotide identity (%)	Amino acid identity (%)	Mutation	Mutation type^a^	Product or function
*gyrA*	OLC2588	99.73	99.17	83- TCC (Ser)→TTC (Phe)	NC	
						DNA gyrase subunit A
	OLC2622	99.73	99.17	83- TCC (Ser)→TTC (Phe)	NC	
	
*nuoF*	OLC2536	99.93	99.78	45- CTG (Leu) → CGC (Arg)	SC	
		
	OLC2556	98.51	99.78	378- CCG (Pro) → TCG (Ser)	SC
						NADH:ubiquinone oxidoreductase (subunit F: the binding site)
	OLC2562	98.43	99.78	257- AAG (Lys) → AGG (Arg)	C	
		
	OLC2563	98.43	99.78	257- AAG (Lys) → AGG (Arg)	C	
	
*prfB*	OLC2619	98.87	99.66	93- GTC (Val) → ATC (Ile)	C	
						Peptide chain release factor 2
	OLC2642	98.87	99.66	93- GTC (Val) → ATC (Ile)	C	
	
	OLC2587	99.31	99.66	242- GCT (Ala) → TCT (Ser)	C	
						Ubiquinone biosynthesis Parahydroxybenzoate ocatprenyltransferase
	OLC2591	99.2	99.66	221- GGC (Gly) → GCC (Ala)	SC	
		
*ubiA*	OLC2593	99.2	99.66	41- CCG (Pro) → TCG (Ser)	NC	
		
	OLC2612	99.2	99.66	41- CCG (Pro) → TCG (Ser)	NC	
		
	OLC2613	99.2	99.66	41- CCG (Pro) → TCG (Ser)	NC	
		
	OLC2625	99.31	99.66	220- CTT (Leu) → TTT(Phe)	C	

*^*a*^Refers to the type of amino acid substitution: NC, non-conservative, residues are not similar; SC, semi-conservative, residues have weakly similar properties; C, conservative, residues have strongly similar properties.*

The performance of AMR detection tools was evaluated by assessing the accuracy of WGS-based predictions relative to the Sensititre Vizion^TM^ phenotype results for each antibiotic. A true positive (TP) was defined as a result where the WGS analysis of an isolate predicted a resistance gene and the strain displayed a resistant phenotype. A false positive (FP) was defined as a result where WGS analysis predicted a resistance gene but the isolate was phenotypically sensitive. A true negative (TN) was defined as a result where WGS analysis predicted no ARGs and the isolate was phenotypically sensitive. A false negative (FN) was defined as a result where WGS analysis did not detect an ARG but the isolate was phenotypically resistant. The accuracy of each tool for each antibiotic was calculated by dividing the sum of TP and TN by the total population (*n* = 111) and multiplied by 100. The overall accuracy for each tool was determined by dividing the sum of TP and TN for all resistances combined divided by the combined number of predictions (*n* = 1332).

### Nucleotide Sequence Accession Numbers

Whole-genome sequences have been deposited at DDBJ/EMBL/GenBank in bioproject PRJNA417863. Sequence read archive (SRA) accession numbers and phenotype data are listed in Supplementary Table S1.

### Raw Read Sampling to Determine Minimum Coverage Requirements for ARG Detection

To determine the minimum genome coverage required for accurate ARG detection, the raw reads for each isolate were randomly subsampled to coverage levels of 1X, 2.5X, 5X, 10X, 15X, and 20X (100 replicates per isolate at each coverage level) using the reformat. sh script (version 37.61) provided with the BBMap suite (Bushnell, 2014). Subsampled reads were analyzed for the presence of AMR genes using the *k*-mer alignment method (KMA v 1.17) (Clausen et al., 2018) with the NCBI-AMR db and default settings. For each isolate, 100 replicates were sampled at each coverage level (*n* = 111 isolates, six coverage levels).

### Analysis of ARG-Detection in Assembled Versus Raw-Read Sequences

Additional subsampling was conducted in order to test the effects of assembly on ARG detection. Raw reads for a subset of seven isolates (Table 3) were randomly subsampled to levels of 5X, 10X, 15X, and 20X coverage as described above (20 replicates per isolate at each coverage level). For each isolate, all replicates at each coverage level were then assembled as described above. ARG detection was conducted using KMA v1.17 with the NCBI-AMR db and default values for both assembled and raw-read sequences. To evaluate statistical significance, comparison of gene-detection in assembled and raw-read sequences was conducted for each gene at each coverage level using the Fisher’s exact test in R version 3.6.1 (R Core Team, 2014).

**TABLE 3:** ARG profiles and locations in subset of *S*. *enterica* subspecies *enterica* isolates used for assembly versus raw-read analyses.

Isolate	Location	Plasmid Inc Type^a^	ARG(s)^b^
OLC2545	Chr		*aac*(6′)-Iaa
	Pmd 960	IncA/C2	*blaCMY*-2, *tetA*, *sul1*, *floR*, *sul2*, *strB* (*aph*(6)-Id), *aadA1*, *strA* (*aph*(3″)-Ib), *aac*(3)
OLC2552	Chr		*fosA7*, *aac*(6′)-Iaa
	Pmd 53	IncX1	−
	Pmd 95	ColRNAI_rep_cluster_1987	−
	Pmd 292	ColRNAI_rep_cluster_1857	−
OLC2564	Chr		*fosA7*, *aac*(6′)-Iaa
	Pmd 473	IncI1	*sul1*, *aac*(3), *aadA1*
	Pmd 695		*tetA*
OLC2568	Chr		*fosA7*, *aac*(6′)-Iaa
	Pmd 53	IncX1	−
	Pmd 60	ColRNAI_rep_cluster_1993	−
	Pmd 61	ColRNAI_rep_cluster_1993	−
	Pmd 357	ColRNAI_rep_cluster_1987	*strA* (*aph*(3″)-Ib), *strB* (*aph*(6)-Id), *dfrA14*, *sul2*
	Pmd 476	IncI1	*blaCMY*-2
OLC2588	Chr		*aac*(6′)-Iaa
	Pmd 476	IncI1	*blaCMY*-2
	Pmd 596	IncX1	−
	Pmd 973	IncFIB, IncFIIA	*strA* (*aph*(3″)-Ib), *strB* (*aph*(6)-Id), *tet*(B)
OLC2604	Chr		*aac*(6′)-Iaa, *aadA2*, *tet*(G), *floR*, *blaCARB, sul1*
	Pmd 34	ColRNAI_rep_cluster1857	*-*
	Pmd 369	IncFIB	−
OLC2643	Chr		*aac*(6′)-Iaa, *catA2*, *fosA7*
	Pmd 53	IncX1	−
	Pmd 61	ColRNAI_rep_cluster_1993	−
	Pmd 476	IncI1	*blaCMY*-2
	Pmd 973	IncFIB, IncFIIA	*strA* (*aph*(3″)-Ib), *sul2*, *blaTEM*, *strB* (*aph*(6)-Id), *dfrA14*, *tetA*

*Abbreviations: ARG, Antimicrobial Resistance Gene; Chr, Chromosome; Pmd, Plasmid; Inc, Incompatibility grouping. ^*a*^Predicted using MOB-suite (https://github.com/phac-nml/mob-suite). Blanks indicate no Inc group was predicted for that plasmid. ^*b*^ARGs were assigned to plasmids or chromosome by matching the contig containing the ARG as reported by ResFinder to the contig output of MOB-suite’s MOB-recon tool.*

### Resistance Phenotype Verification via Broth Microdilution

Discrepancies observed between original AMR genotypes and phenotypes were retested using the broth microdilution method as described by Wiegand et al. (2008). Eleven strains including four strains with genotypic resistance and phenotypic sensitivity, four control strains with genotypic and phenotypic resistance, two strains with a sensitive genotype and phenotypic resistance, and the type strain ATCC 25922 *Escherichia coli* (sensitive control) were tested in sterile 96-well microtiter plates. Antimicrobial concentrations tested included GEN (0.25 – 16 μg/ml), FOX (0.5 – 32 μg/ml), AMC (1/0.5 – 32/16 μg/ml), TCY (1 – 64 μg/ml), and STR (2 – 128 μg/ml). Uninoculated MHB (Oxoid, Nepean, ON, Canada) wells were included as a contamination control. Each of the 11 isolates was inoculated at concentration of approximately 5 × 10^5^ CFU/mL and incubated at 37°C for 24 h. All strains were tested for all antibiotics.

### Streptomycin Sensitivity via Agar Dilution

Streptomycin phenotypic resistance was re-evaluated using the agar dilution method using protocols adapted from Wiegand et al. (2008). Briefly, isolates were streaked for single colonies onto MH agar (MHA) and incubated overnight at 37°C. STR was diluted in MHA at concentrations of 0, 2, 4, 8, 16, 32, and 64 μg/mL. A 0.5 McFarland suspension of each isolate was made then diluted 1:10 in MHB. A 48-pin replicator was used to spot 1 μL aliquots on dried MHA containing STR in duplicate moving from lowest to highest STR concentration in duplicate (0 μg/mL being first as a viability control). *E. coli* ATCC 25922 was included as a sensitive control. All plates were incubated overnight at 37°C. The MIC for each isolate was recorded as the lowest concentration of STR that completely inhibited growth.

### Activation of Cryptic Aminoglycoside Resistance in Minimal Media

To further investigate possible resistance mechanisms for 16 isolates that were resistant to STR, but with no identified ARGs, MICs for STR were evaluated using a method adapted from Koskiniemi et al. (2011). Briefly, MH (Oxoid, Nepean, ON, Canada), Luria-Bertani - Lennox (LB) (Sigma-Aldrich, Oakville, ON, Canada), and M9 minimal salts 5X powder (Sigma-Aldrich, Oakville, ON, Canada) supplemented with 2 mM MgSO_4_, 0.1 mM CaCl_2_, and 0.4% glucose were prepared in both broth and agar forms. Agars contained 0, 2, 4, 8, 16, 32, 64, or 128 μg/ml STR. A 0.5 McFarland suspension was prepared for each isolate in 0.9% saline using a fresh overnight culture, including *E. coli* ATCC 25922 as a control.

For broth microdilution testing using the media described above, the CMV4AGNF Sensititre plate (Trek Diagnostic Systems, Cleveland, OH, United States; Thermo Fisher Scientific, United States) was used to test for resistance to streptomycin (STR) at concentrations of 2, 4, 8, 16, 32, and 64 μg/mL. A 1% stock of TTC (2,3,5-triphenyl-tetrazolium chloride) (Sigma-Aldrich, Oakville, ON, Canada) was mixed with each broth type (M9B, MHB, LB) to create a 0.005% TTC-broth solution. McFarland suspensions were then diluted 1:1000 in each 0.005% TTC-broth type, vortexed, and distributed into the wells of a CMV4AGNF Sensititre plate.

For agar dilution testing, 0.5 McFarland suspensions were diluted 1:10 in 0.9% saline and 2 μl was spotted onto each agar type in duplicate as described above. All agar and broth microtiter plates were incubated at 37°C for 20 h. MIC for each media-antimicrobial combination was recorded as the lowest concentration of antibiotic that led to complete growth inhibition.

## Results

### Determining *Salmonella* spp. Serotypes *in silico*

Both SeqSero2 and SISTR correctly identified most of the isolates (Table 4). For SeqSero2 used with either raw reads or assembled genomes, full matches were observed for 96 serotype predictions (86.5%). SISTR was slightly more accurate, with full matches observed for 98 (88.3%) of the isolates tested. No incorrect results were observed in the dataset used in this study. Neither tool was able to accurately predict the serotype Othmarschen, however, SISTR did report these three isolates as “Haelsingborg| Moers| Oranienburg| Othmarschen” (inconclusive) whereas SeqSero identified these as the closely related Oranienberg serovar. In the latter case results were classified as inconclusive rather than incorrect due to the close relationship of these serovars (Robertson et al., 2018). SeqSero2 generated inconclusive results for Albany and Molade whereas SISTR was able to accurately assign these serovars (Table 5). Incongruent matches were observed for ten SeqSero and SISTR predictions (Table 4). For four of the strains identified as serovar Kentucky by both SISTR and SeqSero2 (OL2571, OLC2572, OLC2573, OLC2621) only some of the antigens were expressed based on traditional serotyping results, even though genes encoding the antigens were detected in the genomes (Supplementary Table S1). A similar situation was observed for isolate OLC2574 (Hadar), OLC2641 (Mbandaka), OLC2616/OLC2640 (Senftenberg) and one of the monophasic variants of *S. enterica* ser. Typhimurium (OLC2556) that was identified as Typhimurium by SeqSero2 and SISTR. One I:Rough-O:R:1,5 isolate (OLC2582), was identified as serovar Infantis by SISTR; however, genes encoding the O-antigen were not identified by SeqSero2 (Supplementary Table S1). Discrepancies between serotype prediction and conventional serotyping were not evaluated by repeating the serotyping.

**TABLE 4:** Performance of *in silico* tools for detecting *Salmonella* serotype.

Match Result	SeqSero2 assembled	SeqSero2 raw reads	SISTR
Full	96 (86.5%)	96 (86.5%)	98 (88.3%)
Inconclusive	5 (4.5%)	5 (4.5%)	3 (2.7%)
Incongruent	10 (9.0%)	10 (9.0%)	10 (9.0%)
Total	111	111	111

*Abbreviations: SeqSero, sequence serotyping tool; SISTR, *Salmonella in silico* typing resource tool. Match Result definitions: Full, serotype prediction concordant with laboratory method; Inconclusive, multiple serotypes were predicted including the laboratory result; Incongruent, results differed because one or more of the antigen genes were not expressed and therefore not detected by laboratory methods.*

**TABLE 5:** Predicting *Salmonella* serotypes using WGS data.

Group	Serotype^a^	Subspecies	Somatic (O) antigens^b^	Flagellar (H) antigens			No. of Isolates	SISTR*		SeqSero2*	
							
				Phase 1	Phase 2	Other^c^		TP	FP	TP	FP
O:1,3,19 (E_4_)	Senftenberg	I	1,3,19	g,[s],t	−	[z_27_],[z_34_],[z_37_], [z_43_],[z_45_],[z_46_], [z_82_]	1	1	2	1	2
	I:Rough-O:g,s,t:-	I	Rough	g,s,t	−		1	0	0	0	0
	I:19:-:-	I	19	−	−		1	0	0	0	0
O:3,10 (E_1_)	Anatum	I	3,{10}{15}{15,34}	e,h	1,6	[z_64_]	1	1	0	1	0
	Orion	I	3,{10}{15}{15,34}	y	1,5		1	1	0	1	0
O:4 (B)	Agona	I	1,4,[5],12	f,g,s	[1,2]	[z_27_],[z_45_]	2	2	0	2	0
	Heidelberg	I	1, 4,[5],12	r	1,2		19	19	0	19	0
	Kiambu	I	1,4,12	z	1,5		5	5	0	5	0
	Saintpaul	I	1,4,[5],12	e,h	1,2		1	1	0	1	0
	Schwarzengrund	I	1,4,12,27	d	1,7		5	5	0	5	0
	Typhimurium	I	1,4,[5],12	i	1,2		12	12	1	12	1
	I:4,5,12:i:-	I	4,5,12	i	−		4	3	0	3	0
O:7 (C_1_)	Braenderup	I	6,7,14	e,h	e,n,z_15_		2	2	0	2	0
	Infantis	I	6,7,14	r	1,5	[R1…],[z_37_],[z_45_], [z_49_]	6	6	1	6	0
	I:Rough-O:r:1,5	I	Rough	r	1,5		1	0	0	0	0
	Mbandaka	I	6,7,14	z_10_	e,n,z15	[z_37_],[z_45_]	3	3	1	3	1
	I:6,7:-:-	I	6,7	−	−		1	0	0	0	0
	Montevideo	I	6,7,14	g,m,[p],s	[1,2,7]		1	1	0	1	0
	Ohio	I	6,7,14	b	l,w	[z_59_]	3	3	0	3	0
	Othmarschen	I	6,7,14	g,m,[t]	−		3	3-I	0	0	0
	Oranienburg	I	6,7,14	m,t	[z_57_]		0	0	0	0	3
	Tennessee	I	6,7,14	z_29_	[1,2,7]		1	1	0	1	0
O:8 (C_2_-C_3_)	Thompson	I	6,7,14	k	1,5	[R1…]	4	4	0	4	0
	Albany	I	8,20	z_4_,z_24_	−	[z_45_]	1	1	0	1-I	0
	Hadar	I	6,8	z_10_	e,n,x		4	4	1	4	1
	I:Rough-O:z_10_:e,n,x	I	Rough	z_10_	e,n,x		1	0	0	0	0
	Kentucky	I	8,20	i	z_6_		9	9	4	9	4
	I:8,20:-:-	I	8,20	−	−		1	0	0	0	0
	I:8,20:I:-	I	8,20	i	−		1	0	0	0	0
	I:8,20:-:z_6_	I	8,20	−	z_6_		1	0	0	0	0
	I:Rough-O:i:z_6_	I	Rough	i	z_6_		1	0	0	0	0
	Litchfield	I	6,8	1,v	1,2		2	2	0	2	0
	Molade	I	8,20	z_10_	z_6_		1	1	0	1-I	0
	Muenchen	I	6,8	d	1,2	[z_67_]	2	2	0	2	0
O:9 (D_1_)	Enteritidis	I	1,9,12	g,m	−		2	2	0	2	0
O:11 (F)	Rubislaw	I	11	r	e,n,x		1	1	0	1	0
O:13 (G)	Cubana	I	1,13,23	z_29_	−	[z_37_],[z_43_]	2	2	0	2	0
	Putten	I	13,23	d	l,w		1	1	0	1	0
	Worthington	I	1,13,23	z	l,w	[z_43_]	1	1	0	1	0
O:35 (O)	Widemarsh	I	35	z_29_	−		1	1	0	1	0
O:40 (R)	Johannesburg	I	1,40	b	e,n,x		1	1	0	1	0
**Total^d^**							111	98	10	96	12

Abbreviations: No., number; SeqSero, sequence serotyping tool; SISTR, *Salmonella in silico* typing resource tool; TP, true positive; FP, false positive. ^*a*^ With the exception of serotype I:4,5,12:i:-, neither tool was able to predict the serotype of isolates designated by a formula (these isolates were missing one or more antigens according to in-lab serotyping). Instead both tools predicted a serotype closely related to the antigenic formula. Formulas are listed below their closest matching serotype. ^*b*^ Underlined O factors are phage-determined epitopes. Rough variants are isolates that do not express an O antigen. ^*c*^ Some isolates express abnormal R phases and/or a third H antigen. R phases agglutinable by anti-1,2 -1,5 -1,6 -1,7 sera but not by anti-2 -5 -6 -7 sera are designated by R1. ^*d*^Inconclusive and incongruent results are not included in totals for TP and FP. *Numbers reported are for analysis of assembled sequences with SISTR and raw-read sequences with SeqSero. Number with adjacent –I flag indicates an inconclusive result where > 1 serotypes were provided by tool. False positive results were incongruent results for closely related serotype with a similar antigenic formula. [ ] = antigenic factors that may be present or absent. ( ) = antigenic factor(s) that are weakly agglutinable. { } = exclusive O-factors (cannot exist with other factors in curly brackets).

### Antimicrobial Resistance: Relationship of Phenotype and Genotype

Four ARG detection tools and four ARG databases were used in seven different combinations (Table 1) to identify at total of 178 ARGs in the 111 *S. enterica* isolates included in this study. With only two exceptions, the ARG tools generated equivalent results (Table 6). KMA analysis of isolate assemblies failed to detect the *dfrA*15 gene for trimethoprim resistance in two isolates using the ResFinder database supplied with the tool. However, KMA analysis of these isolates using the ResFinder database with raw-reads, as well as the NCBI database with assemblies, accurately detected the *dfrA15* gene. The ResFinder tool failed to detect the *sul1* gene for resistance to SOX in a single isolate resulting in 99.1% predictive accuracy. However, the tool was able to detect *sul1* when the select minimum length was lowered to 40%. Further examination revealed that the gene was split between two contigs. This analysis has since been repeated with ResFinder version 3.1 where this split gene was accurately detected, and reported as a > 99% identity for 535/867 bases of Query/Template Length.

**TABLE 6:** Accuracy of AMR phenotype predictions in *Salmonella* by AMR-gene prediction tools.

			No. of test results				
			
		AMR prediction	Phenotype: Sensitive (S)		Phenotype: Resistant (R)		Accuracy (%)
				
Antibiotic	ECV (μg mL^–1^)	Tool*	Genotype:R	Genotype:S	Genotype:R	Genotype:S	
**Aminoglycosides**							
GEN	S: ≤ 4 R: > 16	All	0	100	10	1	99.1
KAN	S: ≤ 16 R: ≥ 64	All	0	110	1	0	100.0
STR	S: ≤ 32 R: ≥ 64	All	13	72	24	2	86.5
STR^*a*^	S: ≤ 16 R: ≥ 32	All	4	72	33	2	94.6
**Beta-lactams**							
AMC	S: ≤ 8 R: ≥ 32	All	0	91	20	0	100.0
**Cephalosporins**							
FOX	S: ≤ 8 R: ≥ 32	All	0	91	20	0	100.0
TIO	S: ≤ 2 R: ≥ 8	All	0	91	20	0	100.0
CRO	S: ≤ 16 R: ≥ 4	All	0	91	20	0	100.0
**Penicillin**							
AMP	S: ≤ 8 R: ≥ 32	All	0	84	27	0	100.0
Phenicol							
CHL	S: ≤ 8 R: ≥ 32	All	0	105	6	0	100.0
**Folate Pathway Inhibitors**							
SOX	S: ≤ 256 R: ≥ 512		0	86	25	0	100.0
		ResFinder v2.1*	0	86	24	1	99.1
SXT	S: ≤ 2 R: ≥ 4		0	103	8	0	100.0
		KMA-Assembled*	0	103	6	2	98.2
**Tetracycline**							
TCY	S: ≤ 4 R: ≥ 16	All	0	80	31	0	100.0

*Except where ResFinder v2.1 and KMA listed separately, all AMR prediction tools (CARD-RGI, srst2 -ResFinder -ARGannot -NCBI, KMA -assemblies and -raw-reads (ResFinder database), and ResFinder-v2.1 and -v3.1 server through center for genomic epidemiology website) performed equally. ^*a*^ Streptomycin testing for ECV ≤ 16 (μg mL^–1^) was conducted using the agar dilution method. All other resistance phenotypes were determined by broth microdilution using the Sensititre system (Trek Diagnostic Systems, Cleveland, OH, United States). ECVs according to CLSI guidelines. Abbreviations: ECV, epidemiological cutoff value; AMR, antimicrobial resistance; ResFinder, Resistance Finder; CARD-RGI, Comprehensive Antibiotic Resistance Database - Resistance Gene Identifier; SRST2, short read sequence typing v0.2.0; NCBI, National Center for Biotechnology Information; KMA, *k*-mer alignment; GEN, gentamicin; KAN, kanamycin; STR, streptomycin; AMC, amoxicillin-clavulanic acid; FOX, cefoxitin; TIO, ceftiofur; CRO, ceftriaxone; AMP, ampicillin; CHL, chloramphenicol; SOX, sulfisoxazole; SXT, trimethoprim-sulfamethoxazole; TCY, tetracycline.

With some exceptions, resistance to antimicrobials in the 58 resistant *S. enterica* strains (including 223 AMR phenotypes) was accurately predicted (> 99%) based on genotype (Table 6). There were originally 17 discrepancies where an ARG was detected yet the isolate was phenotypically sensitive. Repeat testing of isolates OLC2589, OLC2594, OLC2622, and OLC2644 by broth microdilution confirmed WGS-based predictions of FOX; GEN and TCY; FOX; FOX and AMX resistance, respectively (Table 6; Table 7). The remaining 13 isolates were predicted to be STR-resistant but were deemed sensitive based on an epidemiological cutoff value (ECV) of ≥ 64 μg/mL. Due to discrepancies in STR phenotypic and genomic resistance, Tyson et al. (2016) suggested that STR epidemiological cutoff values be lowered to resistance at ≥ 32 μg/mL. All isolates were re-tested for STR-resistance by agar dilution and the reduced ECV ≥ 32 μg/mL was applied. This decreased the number of false positive STR-resistant genotypes from thirteen to four (Table 6 and Supplementary Table S2). Three false negative genotypes in which there were no detected ARGs by any of the tools used in this study yet phenotypic resistance to GEN (OLC2626) or STR (OLC2536 and OLC2644) were observed. Broth microdilution testing of these isolates confirmed original phenotypic testing (Tables 6, 7 and Supplementary Table S2).

**TABLE 7:** Broth microdilution testing of isolates with genotype-phenotype discrepancies.

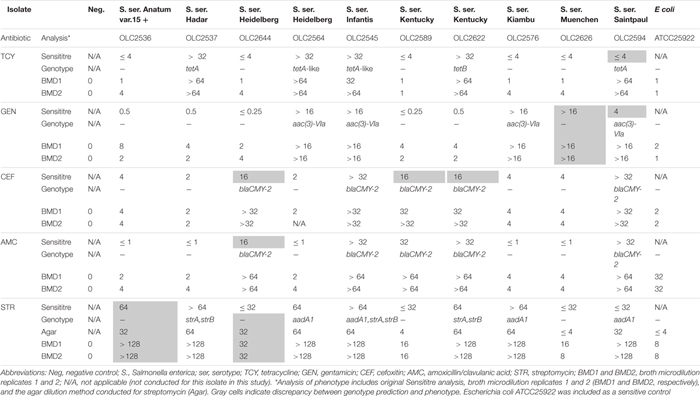	

Following verification of discrepancies with repeat testing, the accuracy of predicting AMR based on ARG detection was determined (Table 6). The accuracy for all tools was > 99% for predicting resistance to aminoglycosides GEN and KAN; β-lactams AMC, FOX, TIO, CRO, and AMP; the phenicol CHL; TCY; and the folate synthesis inhibitor SOX (Table 6). The accuracy of predicting phenotypic AMR to SXT was 100% for all tools except for KMA-analysis of assembled genomes (Table 6). The accuracy for genotypic prediction of phenotypic STR resistance increased from 86.5% to 94.6% when the ECV was lowered from 64 μg/mL to 32 μg/mL.

To determine if ARGs were plasmid- or chromosomally encoded, samples were analyzed with MOB-suite v. 1.4.1 (Robertson and Nash, 2018). Many of the genes were predicted to be plasmid encoded, while some genes were exclusively determined to occur within the chromosomal sequences (*aac(6′)-Iaa*, *fosA7*, Table 3 and Supplementary Table S3). In some cases, ARGs were predicted in both locations (*sul1*, *floR*) (Table 3 and Supplementary Table S3; Robertson and Nash, 2018).

### Minimum Coverage Requirements for Accurate ARG Determination

**FIGURE 1:**
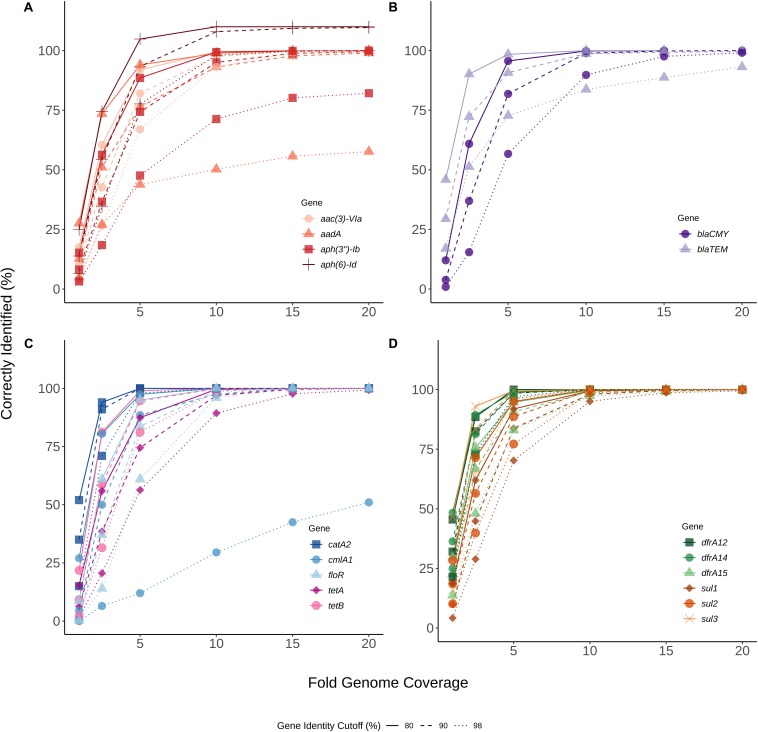
Genome coverage required to detect antibiotic resistance genes (ARGs). Various levels of sequence coverage (1X, 2.5X, 5X, 10X, 15X, 20X) were subsampled 100 times from raw-reads of sequence files for each of 111 *Salmonella* isolates. **(A)** Aminoglycoside resistance genes, **(B)** Beta-lactamase resistance genes, **(C)** Phenicol, florfenicol, and tetracycline resistance genes, **(D)** Trimethoprim and sulphonamide resistance genes. Each of the subsampled sequences was analyzed for ARGs using KMA v 1.17 and the ResFinder database. Percent correctly identified at 80% (continuous line), 90% (dashed line) and 98% (dotted line) gene identity was determined by dividing the total number of hits by the expected number of hits. The *x*-axis represents the sampled fold genome coverage. Genes are differentiated by color and shape (Gene).

To assess sequence coverage requirements for accurate ARG detection, a simulated dataset was analyzed with KMA. Sequence data for each of the 111 *S. enterica* isolates were subsampled to generate sequence coverages ranging from 1X to 20X, with 100 replicates at each coverage level (Figure 1). With a 98% target-gene identity cut-off and 20X genome coverage, the percent of ARGs correctly identified was > 90% for all genes except *ant*(3″)-Ia, and *aadA3* which were detected 73.7% and 70.7%, respectively. At 20X genome coverage with a 90% target-gene identity, ARG detection was > 98% for all genes except *ant*(3″)-Ia, *aadA3*, and *dfrA15b* which were detected in 94.9, 97.7, and 97.7% of the simulated datasets, respectively (Figure 1). At 80% and 90% identity ARGs were accurately identified; however, occasionally alternative alleles were reported for genes *aadA*, *tetA*, and *dfrA*14 (data not shown).

### Effects of Assembly on ARG Detection

**FIGURE 2:**
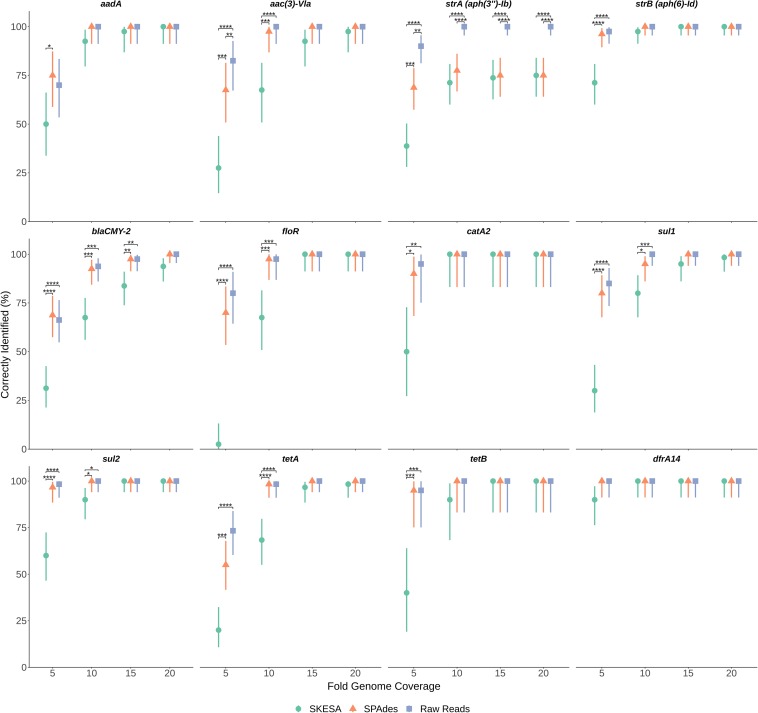
Effects of sequence coverage and assembly on ARG detection. Levels of 5X, 10X, 15X and 20X genome coverage were subsampled 20 times from raw-reads of sequence files for seven *Salmonella* isolates and assembled using both SPAdes and SKESA. Panels are separated by gene (listed at top of each panel). Proportion gene was identified out of *n* trials (*n* = 20, 40, 60, or 80 depending on gene) is plotted on *y*-axis with upper and lower 95% confidence intervals. Significance of proportion detected between assemblies and raw-reads was determined for each gene at each coverage level using Fisher’s exact test. Significance values are displayed above corresponding data points: *p* < 0.05 = *; *p* < 0.01 = **; *p* < 0.001 = ***; *p* < 0.0001 = ****

To determine impact of genome assembly on ARG detection, raw-reads were subsampled from the WGS data of seven isolates at 5X, 10X, 15X, and 20X genome coverages then assembled with both SKESA and SPAdes. Isolates were selected to include various ARG profiles, including a sensitive isolate (Table 3). ARG detection in sub-sampled SKESA and SPAdes assemblies was compared to detection in sub-sampled raw reads at each coverage level using the KMA tool (Table 1 and Figure 2). Significant differences were observed at 5X genome coverage between SKESA assemblies and both SPAdes assemblies and raw-read sequences for all ARGs except *dfrA14* (Figure 2). As coverage increased AMR predictions with either assembly method and raw-reads improved. Compared to SKESA, *blaCMY*-2 was more reliably detected in SPAdes-assembled and raw-read sequence data at 5X, 10X, and 15X genome coverage (Figure 2). Similarly, *aac*(3)-*Vla*, *floR*, *sul1*, *sul2*, and *tetA* were detected at significantly higher proportions in SPAdes assemblies and raw-reads compared to SKESA assemblies at 10X coverage. Overall, *strA* had a lower detection frequency than the other ARGs in assembled genomes. This gene was only detected twice out of 20 replicate assemblies in one isolate (OLC2568). Further investigation of the genome found the *strA* gene among two smaller separated fragments in the assembled genomes. Annotation of the OLC2568 sequence revealed the insertion of the dihydrofolate reductase gene *dfrA14* in the middle of the *strA* coding region. Detection of *strA* was significantly higher at all coverage levels using raw-read sequence data, and SPAdes outperformed SKESA at coverage levels of 5X and 10X. Table 3 depicts the location of the ARGs in the seven test isolates.

### Single Nucleotide Polymorphisms (SNPs) Conferring AMR

The Center for Genomic Epidemiology’s PointFinder program was used to investigate SNPs known to confer resistance to antibiotics (Zankari et al., 2012). Two isolates (OLC2588 and OLC2622) with phenotypic NAL resistance and intermediate ciprofloxacin resistance had SNPs in *gyrA* resulting in non-synonymous mutations at amino acid 83 (Table 2 and Supplementary Table S2); mutations at this position are known to confer quinolone resistance (Ruiz et al., 1997; Piddock et al., 1998; Hakanen et al., 1999; Reche et al., 2002; Cooper et al., 2015, 2016).

To identify the genetic basis of STR and GEN resistance in the three isolates (OLC2536, OLC2644, and OLC2626) with no identified ARGs, SNP analyses were conducted on all isolates in this study to identify mutations in genes that have been associated with increased aminoglycoside resistance. No non-synonymous mutations or truncations were found in genes *glnA*, *ubiE* and *rpsL* in any of the 111 isolates. Multiple non-synonymous mutations were found in *gidB*, *cyoB*, *cyoC*, *trkH*, *ispA*, *nuoE*, *ubiF*, and *aroD* (data not shown), and one non-synonymous mutation was found for *fusA*, yet no associated phenotypic resistance associated with these mutations were observed. A small number of non-synonymous mutations were also observed for *nuoF*, *prfB*, and *ubiA* in a few isolates, some of which could possibly alter function (Table 2).

Comparison of the Nuo protein complex of STR resistant OLC2536 to *S. enterica* ser. Anatum var. 15 + genomes in the OLC-CFIA collection as well as a publicly available STR sensitive *S. enterica* ser. Anatum var. 15 + genome (Accession: NZ_CP013222) revealed a SNP in the *nuoF* coding region. This resulted in a non-synonymous 45-CTG (Leu) → CGG (Arg) mutation. Further analysis of the aligned *nuoF* region using the NCBI blast database found the 45-CTG codon to be highly conserved in the *nuoF* region of aligned *S. enterica* genomes (all results exhibited 99% identity to OLC2536 in this study). This gene was highly conserved among all isolates tested with only four of 111 isolates exhibiting < 100% amino acid identity to the sensitive reference. Out of the four isolates only OLC2536 exhibited a L45R substitution while three other isolates had P378S (*n* = 1) or K257R (*n* = 2) substitutions (Table 2). Of the isolates with non-synonymous mutations in *nuoF*, only OLC2536 presented with phenotypic STR resistance. Two other *S. enterica* ser. Anatum isolates (one confirmed var. 15 +) in the CFIA collection also harbored the L45R mutation with no other ARGs and were phenotypically STR-resistant (data not shown). Two more distantly related *S. enterica* ser. Anatum isolates (not var. 15+, approximately 160 SNP difference with OLC2536) which encoded leucine at codon 45 of *nuoF* and did not harbor any STR-resistance genes were phenotypically STR-sensitive.

Comparison of *S. enterica* ser. Heidelberg isolates OLC2644 (STR resistant) and OLC2552 (STR sensitive) found ten SNPs. SNPs were located in the DUF3626 domain-containing protein (accession: WP_000917268.1), the phosphoenolpyruvate-dependent sugar phosphotransferase system (PTS) galactitol-specific EIIC component *gatC* (accession: WP_000460837.1), L-cystine-binding protein *fliY* (accession: WP_000949370.1), *tetR*/*acrR* family transcriptional regulator (accession: WP_000208474.1), sensor kinase *dpiB* (accession: ACF66636.1), *yjjI* family glycine radical enzyme (accession: WP_001111688.1), nickel/cobalt transporter (accession: WP_000111019.1), a hypothetical protein (accession: WP_107321080.1), and an uncharacterized genomic region. Non-synonymous mutations were located in *citA* (Q324L), *yjjI* (L499Q), and a nickel/cobalt transporter (G51D) of OLC2644. A nonsense mutation also occurred in *fliY* of OLC2644 at codon 202.

Comparison of *S. enterica* ser. Muenchen isolates OLC2626 (GEN resistant) and OLC2592 (GEN sensitive) resulted in three SNPs. SNPs were located in the bifunctional glycosyltransferase/transpeptidase penicillin binding protein 1 gene *mrcB* (accession: WP_052934909.1), phosphate inducible starvation protein gene *psiE* (accession: WP_000982749.1), and a DUF1176 domain-containing protein of unknown function (accession: WP_001270678.1). None of these SNPs resulted in non-synonymous mutations. OLC2592 also harbored a plasmid encoding resistance to sulphonamides (*sul2*), tetracycline (*tetA*), and streptomycin (*strA* and *strB*).

### Minimal Media Induces Cryptic Aminoglycoside Resistance in *Salmonella* spp. Isolates

To study the impact of minimal media (M9) on STR resistance, a subset including both STR sensitive and resistant isolates was tested by broth and agar microdilution (Table 8). Differences were observed for STR MICs in broth compared to agar for all isolates tested. All isolates grew on all agar formulations without STR, except OLC2542 which exhibited limited growth on M9. Increased STR MICs were observed both in M9 and LB agar and broth compared to MH for most isolates, even those characterized as STR sensitive (Table 8). With the exception of OLC2542 and *E. coli* ATCC 25922 (sensitive control), all isolates exhibited extremely high MICs (≥ 64 μg/ml) in M9 media (Table 8).

**TABLE 8:** STR minimum inhibitory concentration of *Salmonella* isolates in MH, LB, and M9 agar and broth.

Isolate	Serotype	Aminoglycoside resistance gene(s)	MIC Streptomycin (μg/ml)^*a*^					
			
			Agar			Broth		
				
			MH	LB	M9	MH	LB	M9
OLC2536	Anatum var. 15 +		64	> 128	>128	> 64	>64	> 64
OLC2540	Heidelberg	*aph*(3′)-Ia	8	32	> 128	16	64	> 64
OLC2541	Heidelberg	*aadA1*-like,*strA*,*strB*	> 128	>128	> 128	>64	> 64	>64
OLC2542	Heidelberg	*aac*(3)-VIa-like,*aadA1*	64	> 128	LG	> 64	>64	LG
OLC2548	Ohio	*aadA1*	32	128	> 128	>64	> 64	>64
OLC2560	Ohio		4	8	128	16	16	> 64
OLC2561	Ohio		8	16	128	16	32	> 64
OLC2568	Heidelberg	*strA*-like, *strB*-like	8	32	> 128	16	64	> 64
OLC2575	Kiambu	*strA*-like, *strB*	8	32	> 128	16	64	> 64
OLC2576	Kiambu	*aac*(3)-VIa-like,*aadA1*	64	> 128	>128	> 64	>64	> 64
OLC2577	Kiambu		8	32	> 128	32	32	> 64
OLC2592	Muenchen	*strA*, *strB*-like	32	128	> 128	>64	> 64	>64
OLC2596	Thompson	*aadA1*, *aadA2*	16	64	> 128	32	> 64	>64
OLC2597	Thompson	*aadA1*, *aadA2*	16	64	> 128	>64	> 64	>64
OLC2626	Muenchen		4	8	64	8	16	> 64
OLC2634	Thompson		8	16	> 128	NT	NT	NT
ATCC25922	*E. coli*	negative control	4	8	4	8	16	8

*Abbreviations: STR, streptomycin; AMR, antimicrobial resistance; MIC, minimum inhibitory concentration; MH, Mueller Hinton; LB, Luria-Bertani Lennox formulation; M9, minimal salts media; NT, not tested; LG, limited growth (no growth after 24 h). ^*a*^MIC of ≥ 32 μg/ml in MH is considered resistant in Mueller Hinton media.*

## Discussion

*Salmonella* spp. colonize a range of animal hosts; consequently, in industrialized countries, the majority of human infections are associated with contaminated animal food products (Butaye et al., 2006). Specific serotypes and AMR profiles can be linked to food commodities which can vary depending on antimicrobial usage for food production in different countries (Butaye et al., 2006; Zhao et al., 2007; Hong et al., 2016; Yoon et al., 2017). As such, the resistance profile and serotype of a *Salmonella* isolate can provide clues as to the epidemiology of an infection (Pornsukarom et al., 2018). Some examples include MDR *S. enterica* ser. Newport associated with exposure to dairy cattle and beef in the United States (Holmberg et al., 1984; Centers for Disease Control and Prevention [CDC], 2002; Butaye et al., 2006; Schneider et al., 2011; Plumb, 2019), MDR *S. enterica* ser. Heidelberg is frequently associated with poultry in both Canada and the United States (Dutil et al., 2010; Routh et al., 2015; Gieraltowski et al., 2016), and MDR *S. enterica* ser. Paratyphi B variant Java has been linked to poultry in Europe (van Pelt et al., 2003; Threlfall et al., 2005; Butaye et al., 2006). The association between AMR phenotype and serotype could provide valuable clues as to the possible source of infection for risk assessment and epidemiological investigations.

Genome-based prediction of *Salmonella* serotype and AMR is increasingly being used by public-health organizations worldwide (Inouye et al., 2014; Bradley et al., 2015; Licker et al., 2015; Tyson et al., 2015, 2016; Clausen et al., 2016; McDermott et al., 2016; Zhao et al., 2016). These predictions are conducted using a variety of computational algorithms which rely on different databases, with few comparative analyses of approaches. We found that AMR and serotype could be accurately predicted in *S. enterica* from WGS data using several widely available programs with minimal differences. While approaches for genoserotyping rely on similar antigen markers (Yachison et al., 2017; Uelze et al., 2020), for AMR there are currently multiple databases containing lists of known resistance genes/mutations, with most databases focusing on acquired ARGs with implications in human and veterinary medicine (Inouye et al., 2014; Bradley et al., 2015; Licker et al., 2015; Tyson et al., 2015, 2016; Clausen et al., 2016; McDermott et al., 2016; Zhao et al., 2016; Feldgarden et al., 2019). The phenotype prediction tools tested in this study provided similar results with minimal variation. Variability observed in this study can be explained by differences among databases, application of computational algorithms, or difficulties in detection of AMR resulting from point mutations. Overall WGS analysis was more reliable than phenotyping as it identified several discordant results that were corrected upon retesting.

### Reliability of *Salmonella* Serotyping Tools

SeqSero2 and SISTR determine *Salmonella* serotypes from WGS data based on matches to genes encoding somatic and flagellar antigens (Zhang et al., 2015, 2019; Yoshida et al., 2016). The *in silico* tools then use the predicted antigenic formula to determine the most likely named serotype in the Kauffmann-White-Le Minor scheme (Grimont and Weill, 2007; Table 5 and Supplementary Table S1). Both of these tools performed well for serotype determination for the 111 isolates included in this study, with unambiguous identification of serovars for over 88.3 and 86.5% of isolates using SISTR and SeqSero2, respectively, and no “incorrect” serotype identification (Table 4). As in previous studies, SISTR performed slightly better than SeqSero2 for resolving “inconclusive” results due to use of cgMLST for distinguishing serovars with the same antigenic profile (Table 4 and Supplementary Table S1; Yachison et al., 2017; Uelze et al., 2020). However, both tools generated “inconclusive” results for the three *S. enterica* ser. Othmarshen isolates included in this study. This difficulty distinguishing between serovars Othmarshen and Oranienberg has been described by Robertson et al. (2018). The authors provide evidence that these two serovars are not genetically distinct and therefore not easily resolved using *in silico* serotyping tools. “Incongruent” results were observed for ten of the isolates included in this study where genes encoding antigens were detected in WGS data, but were not expressed based on serotyping results. Yachison et al. (2017) suggest a need to carry out further analyses using traditional serotyping for incongruent results and to “reframe serotyping for genomics,” as genes that are carried by an isolate are not necessarily expressed. Conversely, we have also observed cases where presence of a second, plasmid-encoded, flagellar operon masked the detection of the strain’s endogenous flagella, confounding serotyping results (Robertson et al., 2019).

The performance of tools for *in silico Salmonella* serotyping has been extensively evaluated elsewhere. For example, Yachison et al. (2017) and Uelze et al. (2020) evaluated SISTR, SeqSero and Multilocus Sequence Typing (MLST) with 813 and 1624 *Salmonella* isolates, respectively, and Robertson et al. (2018) evaluated accuracy of serotype prediction using SISTR and MLST using 42400 genomes deposited in the sequence read archive (SRA). Yachison et al. (2017) reported unambiguous serotype determination of 89.7% of isolates with SISTR, but only 54.1% of isolates using SeqSero (version 1). In this study, authors considered “inconclusive” and “incongruent” matches to be successful, increasing performance scores to 94.8, 88.2, and 88.3% of the isolates tested using SISTR, SeqSero1, and MLST, respectively. Uelze et al. (2020) report accuracies for unambiguous serovar identification of 94, 87, 81, and 79% for SISTR, SeqSero2, SeqSero1, and MLST, respectively. Higher accuracies in the Uelze et al. study may be due to corrections resulting from repeated serological analyses for isolates where *in silico* predictions were incongruent with initial serotypes (Uelze et al., 2020). Finally, in the large-scale study, conducted by Robertson et al., unambiguous matches were found for 91.9% and 87.5% of isolates using SISTR and MLST, respectively. These studies not only used much larger data sets for their comparisons but also included a number of serotypes not found in our study. Furthermore, the Uelze et al. (2020) and Robertson et al. (2018) studies included *S. enterica* subspecies II to IV in their analyses.

### Prediction of AMR Based on WGS

Due to the increasing importance of AMR surveillance, numerous computational approaches and databases are currently being applied for *in silico* prediction of AMR based on WGS data, and these tools are continually improving and evolving. We evaluated seven combinations of tools and databases (Table 1) and found that all performed equally well with accuracies of ≥ 99% for most tool-database combinations for the set of *S. enterica* investigated in this study, except for the prediction of SXT resistance using KMA which had an accuracy of 98.2%, and the prediction of streptomycin resistance that had an overall accuracy of 94.6% using all computational tools (Table 6). We were unable to determine why KMA with ResFinder database and assembled genomes provided a false negative result for *dfrA15* in these isolates as the same gene/allele is present in both the ResFinder and NCBI databases. In addition, CARD-RGI was also able to detect *dfrA15* in these assembled genomes. Analysis of assemblies using KMA with the NCBI AMR database detected these genes in the three isolates, as did analysis of raw-read data using KMA and SRST2. With the exception of *dfrA15*, we did not observe further differences in performance among resistance gene databases, likely due to the extensive overlap among them, nor with assembly independent versus assembly dependent analyses.

The Comprehensive Antibiotic Resistance Database – Resistance Gene Identifier and the ResFinder WebTool were accessible through web interfaces using databases provided with the tools (McArthur et al., 2013; Zankari, 2014). Use of the SRST2 and KMA tools enabled more flexibility in database selection. Where SRST2 requires specific database formatting as per the developers’ instructions, KMA allows very fast database indexing without requiring clustering and specific header re-formatting. The CARD-RGI results were more extensive than the other tools as they also included multiple hits for efflux pumps and membrane channel proteins that have been found to confer resistance to some antibiotics. These proteins are often chromosomally encoded, typically involved in normal cellular functions, require additional genes and regulators to function, and may be species specific. Thus, the presence of these genes may not be informative for the surveillance of acquired ARGs, and may require additional expertise for data interpretation.

### Requirements for WGS-Based ARG Detection

There is limited discussion in the literature as to the sequence quality and genome coverage required to accurately detect ARGs in WGS data. Poor sequence quality and low coverage could result in assembly artifacts and fragmentation of sequence data. ARG-detection tools requiring assembled genomes risk missing a gene if it is split over multiple contigs (Clausen et al., 2016). Conversely, approaches using Bowtie2 for analyses of raw-read data risk reporting false positives due to contaminating agents in addition to cases where a gene may be fragmented due to insertion of another gene (Clausen et al., 2016).

Using a cutoff of 98% for target-gene identity, ARGs were not always detected at 20X genome coverage (Figure 1). However lowering target-gene identity to the default cutoff of 90%, currently suggested for most *in silico* ARG detection programs, allowed for detection of closely related and novel alleles resulting in 100% gene identification at 15 and 20X for most genes (Figure 1). Some of the aminoglycoside genes were correctly identified at > 100% of expected for coverage of 5 to 20X (Figure 1A). This is likely due to multiple isolates (*n* = 10) encoding multiple alleles and/or copies of the gene at ≥ 80% identity, thereby resulting in a higher number of positive hits (confirmed using KMA on raw-reads, data not shown). In contrast, lower percent identification sometimes occurred for genes that matched closely to multiple alleles, as KMA uses a scoring scheme in order to ensure the best matching template is selected and prevent reporting of false positives, which may have resulted in *k*-mer matches to alternate alleles and under-reporting of genes at lower coverage levels (Figure 1; Clausen et al., 2018). These results suggest a minimum coverage requirement of 15-20X for bacterial isolate WGSs for accurate AMR predictions, and that deeper sequencing in conjunction with lower gene identity cutoffs may improve ARG detection. In addition, ARG analysis of novel or rare bacterial species or strains via WGS may benefit from altering gene identity cutoffs in order to detect new alleles and closely related or novel resistance genes.

We considered the possibility that ARG detection may be more sensitive using raw-read sequences as this would alleviate errors arising from repeat regions and assembly of contaminating agents impacting genome assembly as has been observed in other studies (Carrillo et al., 2016; Clausen et al., 2016; Low et al., 2019). Assembly tools were found to have an impact on the ability to detect ARGs, particularly at low genome coverage where percentages of correctly identified genes were significantly lower in SKESA-assemblies. At higher coverage levels the use of raw-reads and both assembly types for ARG detection gave similar results for all genes tested (Figure 2). In contrast to SPAdes, SKESA is designed to be more conservative, producing assemblies with high base level accuracy and avoiding assembly of potentially questionable sequences (Souvorov et al., 2018).

In the coverage-sampled assembled dataset, the streptomycin resistance gene *strA* was only detected in 10% of OLC2568 assemblies but found in most of the raw-read files (Table 3 and Figure 2). These results suggest identification of non-functional genes is more likely to occur when using raw-read sequence data for gene detection. In this case the fragmentation of *strA* by an inserted *dfrA14* gene in isolate OLC2568 did not affect AMR phenotype predictions as this isolate also harbored a full-length *strB* phosphotransferase.

Overall, ARG detection accuracy for isolate sequences appears to depend on sequence quality and the gene being investigated. Furthermore, if sequence coverage is greater than 15X, assembly methods have minimal impact on ARG detection (Figure 2).

### Single Nucleotide Polymorphisms (SNPs) Conferring AMR

Antimicrobial resistance can be achieved through both acquisition of resistance-conferring genes and genetic adaptation through mutations (Nishimura et al., 2007; Okamoto et al., 2007; Wong et al., 2011; Mikheil et al., 2012; Lázár et al., 2013; Blair et al., 2015). Amino acid substitutions resulting in NAL resistance (NalR) have been well documented in *Salmonella* and *E. coli* (Ruiz et al., 1997; Piddock et al., 1998; Hakanen et al., 1999; Reche et al., 2002; Ruiz, 2003; Cooper et al., 2015, 2016; Knowles et al., 2015). Consistent with the literature, we identified two isolates with non-synonymous mutations in the *gyrA* gene (Table 2) which correlated with observed phenotypic NAL and intermediate CIP resistance in these isolates (Supplementary Table S2). Both isolates harbored non-synonymous mutations at serine 83, which is known to be important for quinolone resistance (Piddock et al., 1998; Ruiz, 2003).

All of the ARG prediction tools failed to detect genes conferring streptomycin resistance in two strains and gentamycin resistance in one strain (Tables 6, 7, and Supplementary Table S2). The three main mechanisms of aminoglycoside resistance include antimicrobial inactivation by aminoglycoside modifying enzymes, ribosome modification, and decreased membrane permeability (Lázár et al., 2013). Mutations resulting in lack of methylation of the 16S rRNA have been found to result in STR resistance in *E. coli*, *Mycobacterium tuberculosis, Bacillus subtilis*, and *Salmonella* spp. (Nishimura et al., 2007; Okamoto et al., 2007; Wong et al., 2011; Mikheil et al., 2012). This loss of methylation has been associated with mutations and/or deletions in the ribosomal small subunit methyltransferase G gene *rsmG* (formerly *gidB*) (Nishimura et al., 2007; Okamoto et al., 2007; Wong et al., 2011; Mikheil et al., 2012) which were not observed in the resistant OLC2536, OLC2626, or OLC2644.

Failure to predict aminoglycoside resistance may also be due to mutations within efflux-related proteins that have not yet been documented (Lázár et al., 2013; Garneau-Tsodikova and Labby, 2016). Comparison of phenotypically resistant *S. enterica* ser. Muenchen (OLC2626) and resistant *S. enterica* ser. Heidelberg (OLC2644) to phenotypically sensitive isolates of the same serovars found a low number of SNPs. Although a few non-synonymous mutations and a nonsense mutation were detected in comparison of the Heidelberg isolates, no obvious cause for STR-resistance in this isolate could be determined.

A study on the evolution of antibiotic hypersensitivity in *E. coli* conducted by Lázár et al. (2013) reported 44% of collateral-sensitivity interactions involved resistance to aminoglycosides. Genetic analyses of hypersensitive mutants identified genes involved in membrane potential including the respiratory electron transport chain (ETC) Nuo (NADH:ubiquinone oxidoreductase) protein complex (Lázár et al., 2013). This is not surprising as aminoglycosides require respiration for uptake and aminoglycoside resistance has been linked to decreased membrane permeability (reviewed by Garneau-Tsodikova and Labby, 2016). Of the isolates in this study with non-synonymous mutations in *nuoF* only the *S. enterica* ser. Anatum var 15 + strain (OLC2536) and *S. enterica* ser. Anatum isolates from the OLC-CFIA culture collection with L45R mutations presented with phenotypic STR resistance (Table 2). Collectively this suggests a role for the *nuoF* L45R mutation in STR-resistance. Further investigation of this mutation in *nuoF* is currently being conducted to determine whether membrane potential and efflux activity are reduced and if the detected SNP in *nuoF* plays a role in this decreased potential and STR resistance.

Blocking of the ubiquinone biosynthesis pathway results in a defect in electron transport and aerobic respiration which has been found to increase aminoglycoside resistance (Paradise et al., 1998; Li et al., 2016). Mutations in *ubiF* have been shown to produce pleiotropic *E. coli* phenotypes resistant to STR and GEN (Soballe and Poole, 1999). Similarly, Li et al. (2016) found mutations in genes *ubiE* and *prfB*, associated with STR induction of a small colony variant (SCV) phenotype, resulted in two- to four- fold increases in STR MIC of isolates. BLAST analyses of the coenzyme Q redox gene *ubiE* found no frameshift or non-synonymous mutations in isolates from this study. Multiple non-synonymous mutations were detected in *ubiF*, and two isolates exhibited V165I mutations in *prfB* (Table 2); however, no obvious change in MIC was observed to result from these mutations.

Frequently, identification of SNP-based resistance requires alignment of protein sequences for the identification of non-synonymous mutations in regions of interest. In cases of novel SNP-based resistance, comparison to closely related isolates may enable identification of resistance-conferring mutations. This is not always possible when a closely related isolate is unavailable. Additional investigations into SNP-based mutations that result in evolution of AMR would be useful not only for determining novel SNP-based resistances, but also classes of genes that are associated with the evolution of AMR (Lázár et al., 2013). Continuing research is needed to identify new genetic factors conferring resistance. The development of more comprehensive curated databases of SNP-based mutations conferring AMR in different pathogenic bacterial species will enable more reliable detection of SNP-based AMR in WGS datasets.

### Activation of STR Resistance in *Salmonella* spp. Occurs in Minimal Media

Similar to the results of Tyson et al. (2016) and Pornsukarom et al. (2018), discrepancies were observed for genotypic prediction of STR resistance even after broth and agar microdilution testing. A study by Koskiniemi et al. (2011) found activation of a chromosomally encoded adenyl transferase (*aadA*) combined with mutations affecting the electron transport chain (ETC) resulted in increased STR resistance. Their work showed that while growth in rich medium (such as LB or BHI) resulted in a phenotypically sensitive isolate, growth in minimal media or mutations that impaired the ETC resulted in conversion to a small colony variant (SCV) and activation of the chromosomal *aadA* gene conferring STR resistance. The subset of *S. enterica* isolates tested in MH, M9, and LB media all exhibited an extremely high resistance to STR in M9 with the exception of OLC2542 which appeared to have impaired growth in minimal media (Table 8). Growth of STR-resistant OLC2536 in M9 was similar to both STR -sensitive and -resistant comparator isolates. However even without harboring acquired STR-resistance genes OLC2536 exhibited growth at high concentrations of STR in rich media (LB and MH), comparable to other STR-resistant isolates, suggesting that ETC mutations in this strain may be conferring STR-resistance as described by Koskiniemi et al. (2011).

## Conclusion

Relationships between *Salmonella* serotype and AMR profile can indicate the possible source of an isolate and may be valuable for epidemiological and outbreak investigations. While identification and resistance determination of bacteria is critical for guiding therapeutic approaches in treating infections, use of genomic approaches has the added benefit of providing data for surveillance purposes. We have shown here that *in silico* tools predicting *Salmonella* serotypes and AMR-phenotypes are highly accurate. In fact, in this study genomic prediction of AMR was more accurate than phenotypic results. Similarly, genome-based serotype determination may be more informative than laboratory approaches for clustering genetically related isolates, particularly in cases where somatic and flagellar antigens are not expressed. However, there are some caveats – namely the importance of sequence coverage and assembly method, the involvement of chromosomal SNPs in mutations conferring resistance, and the role of the environment on resistant phenotypes as this could impact expression of genes conferring resistance. A *nuoF* mutation amongst STR-resistant *S. enterica* ser. Anatum var. 15 + strains was noted; however, the precise mechanism of aminoglycoside-resistance in three strains with no identifiable ARGs remains uncertain and indicates continuing research is needed to catalog the molecular basis of resistance mechanisms. Development and curation of high quality, verified datasets is critical for assessing performance of new pipelines/tools for WGS-analysis of pathogens. This study provides an easily accessible, verified *S. enterica* data set containing both sensitive and resistant isolates of different serotypes for validation of *in silico* tools for both serotype- and AMR-determination.

## Data Availability Statement

The datasets analyzed for this study can be found in the sequence read archive (SRA) under bioproject PRJNA417863 (https://www.ncbi.nlm.nih.gov/bioproject/PRJNA417863). Sensititre phenotype data, MOB-suite plasmid predictions, biosample (SAMN) and compressed SRA file (SRR) identifiers are listed in Supplementary Data (Supplementary Tables S1–S3).

## Author Contributions

AC, CC, and BB conceived and designed the experiments. AC performed laboratory experiments and performed statistical analysis. AC and AL performed *in silico* experiments. AC, CC, AL, AK, and MT analyzed the data. CC, BB, and DL contributed reagents, materials, analysis tools. AC and CC wrote the first draft of the manuscript. AL, AK, BB, AW, and ST contributed to writing of the manuscript. All authors contributed to manuscript revision, read and approved the submitted version.

## Conflict of Interest

The authors declare that the research was conducted in the absence of any commercial or financial relationships that could be construed as a potential conflict of interest.

## Footnotes

1 https://github.com/katholt/srst2

## Supplementary Material

DATA SHEET S1 Sequence and phenotype data for 111 Salmonella isolates. (Table 1) Serovar data, sequence metrics, Sequence Read Archive and SAMN biosample identifiers. (Table 2) Antimicrobial resistance phenotype and genotype data. (Table 3) MOB-suite plasmid predictions.

## References

[B1] Andrews S. (2010). *FastQC: A Quality Control Tool for High Throughput Sequence Data.* Available online at: https://www.bioinformatics.babraham.ac.uk/projects/fastqc/ (accessed September 28, 2018).

[B2] Aurass P. Düvel J. Karste S. Nübel U. Rabsch W. Flieger A. (2017). *glnA* truncation in *Salmonella enterica* results in a small colony variant phenotype, attenuated host cell entry, and reduced expression of flagellin and SPI-1 associated effector genes. *Appl. Environ. Microbiol.* 84:e01838-17. 10.1128/AEM.01838-17 29150501 PMC5752853

[B3] Bankevich A. Nurk S. Antipov D. Gurevich A. A. Dvorkin M. Kulikov A. S. (2012). SPAdes: a new genome assembly algorithm and its applications to single-cell sequencing. *J. Comput. Biol.* 19 455–477. 10.1089/cmb.2012.0021 22506599 PMC3342519

[B4] Blair J. M. A. Webber M. A. Baylay A. J. Ogbolu D. O. Piddock L. J. V. (2015). Molecular mechanisms of antibiotic resistance. *Nat. Rev. Microbiol.* 13 42–51. 10.1038/nrmicro3380 25435309

[B5] Boerlin P. Travis R. Gyles C. L. Reid-Smith R. Heather Lim N. J. Nicholson V. (2005). Antimicrobial resistance and virulence genes of *Escherichia coli* isolates from swine in Ontario. *Appl. Environ. Microbiol.* 71 6753–6761. 10.1128/AEM.71.11.6753-6761.2005 16269706 PMC1287655

[B6] Bopp D. J. Baker D. J. Thompson L. Saylors A. Root T. P. Armstrong L. (2016). Implementation of *Salmonella* serotype determination using pulsed-field gel electrophoresis in a state public health laboratory. *Diagn. Microbiol. Infect. Dis.* 85 416–418. 10.1016/j.diagmicrobio.2016.04.023 27220605

[B7] Bradley P. Gordon N. C. Walker T. M. Dunn L. Heys S. Huang B. (2015). Rapid antibiotic-resistance predictions from genome sequence data for *Staphylococcus aureus* and *Mycobacterium tuberculosis*. *Nat. Commun.* 6:10063. 10.1038/ncomms10063 26686880 PMC4703848

[B8] Bushnell B. (2014). *BBMap: A Fast, Accurate, Splice-Aware Aligner.* Berkeley, CA: Lawrence Berkeley National Laboratory.

[B9] Butaye P. Michael G. B. Schwarz S. Barrett T. J. Brisabois A. White D. G. (2006). The clonal spread of multidrug-resistant non-typhi *Salmonella* serotypes. *Microbes Infect.* 8 1891–1897. 10.1016/j.micinf.2005.12.020 16714135

[B10] Camacho C. Coulouris G. Avagyan V. Ma N. Papadopoulos J. Bealer K. (2009). BLAST+: architecture and applications. *BMC Bioinformatics* 10:421 10.1186/1471-2105-10-421 PMC2803857 20003500

[B11] Cano D. A. Pucciarelli M. G. Martínez-Moya M. Casadesús J. García-del Portillo F. (2003). Selection of small-colony variants of *Salmonella enterica* serovar typhimurium in nonphagocytic eucaryotic cells. *Infect. Immun.* 71 3690–3698. 10.1128/IAI.71.7.3690-3698.2003 12819049 PMC161971

[B12] Carrillo C. D. Koziol A. Vary N. Blais B. W. (2019). “Applications of genomics in regulatory food safety testing in Canada,” in *New Insight into Brucella Infection and Foodborne Diseases*, ed. Ranjbar M. (Tehran: Iran University of Medical Sciences). 10.5772/intechopen.86063

[B13] Carrillo C. D. Koziol A. G. Mathews A. Goji N. Lambert D. Huszczynski G. (2016). Comparative evaluation of genomic and laboratory approaches for determination of Shiga toxin subtypes in *Escherichia coli*. *J. Food Prot.* 79 2078–2085. 10.4315/0362-028X.JFP-16-228 28221953

[B14] Centers for Disease Control and Prevention [CDC] (2002). Outbreak of multidrug-resistant *Salmonella* newport–united states, January-April 2002. *MMWR Morb. Mortal. Wkly. Rep.* 51 545–548. 12118534

[B15] Chan K.-G. (2016). Whole-genome sequencing in the prediction of antimicrobial resistance. *Expert Rev. Anti Infect. Ther.* 14 617–619. 10.1080/14787210.2016.1193005 27215476

[B16] Clausen P. T. L. C. Aarestrup F. M. Lund O. (2018). Rapid and precise alignment of raw reads against redundant databases with KMA. *BMC Bioinformatics* 19:307. 10.1186/s12859-018-2336-6 30157759 PMC6116485

[B17] Clausen P. T. L. C. Zankari E. Aarestrup F. M. Lund O. (2016). Benchmarking of methods for identification of antimicrobial resistance genes in bacterial whole genome data. *J. Antimicrob. Chemother.* 71 2484–2488. 10.1093/jac/dkw184 27365186

[B18] Clinical and Laboratory Standards Institute (2008). *Performance Standards for Antimicrobial Disk and Dilution Susceptibility Tests for Bacteria Isolated from Animals; M31-A3*, 3rd Edn Wayne, PA: Clinical and Laboratory Standards Institute.

[B19] Clinical and Laboratory Standards Institute (2013). *Performance Standards for Antimicrobial Susceptibility Testing; M100-23*, 23rd Edn Wayne, PA: Clinical and Laboratory Standards Institute.

[B20] Cooper A. Koziol A. G. Carrillo C. D. Lambert D. (2016). Draft genome sequences of *Salmonella enterica* subsp. *enterica* serovar berta ATCC 8392 and a nalidixic acid-resistant isolate of this strain. *Genome Announc.* 4:e00186-16. 10.1128/genomeA.00186-16 27103707 PMC4841122

[B21] Cooper A. Lambert D. Koziol A. G. Seyer K. Carrillo C. D. (2015). Draft genome sequence of *Salmonella enterica* subsp. *enterica* serovar mishmarhaemek isolated from bovine feces. *Genome Announc.* 3:e01210-15. 10.1128/genomeA.01210-15 26472847 PMC4611699

[B22] Doyle R. M. O’Sullivan D. M. Aller S. D. Bruchmann S. Clark T. Coello Pelegrin A. (2020). Discordant bioinformatic predictions of antimicrobial resistance from whole-genome sequencing data of bacterial isolates: an inter-laboratory study. *Microb. Genom.* 6:e000335. 10.1099/mgen.0.000335 32048983 PMC7067211

[B23] Dutil L. Irwin R. Finley R. Ng L. K. Avery B. Boerlin P. (2010). Ceftiofur resistance in *Salmonella enterica* serovar Heidelberg from chicken meat and humans, Canada. *Emerg. Infect. Dis.* 16 48–54. 10.3201/eid1601.090729 20031042 PMC2874360

[B24] Edirmanasinghe R. Finley R. Parmley E. J. Avery B. P. Carson C. Bekal S. (2017). A whole-genome sequencing approach to study cefoxitin-resistant *Salmonella enterica* serovar Heidelberg isolates from various sources. *Antimicrob. Agents Chemother.* 61:e01919-16. 10.1128/AAC.01919-16 28137797 PMC5365727

[B25] Feldgarden M. Brover V. Haft D. H. Prasad A. B. Slotta D. J. Tolstoy I. (2019). Validating the NCBI AMRFinder tool and resistance gene database using antimicrobial resistance genotype-phenotype correlations in a collection of NARMS isolates. *Antimicrob. Agents Chemother.* 63:e00483-19. 10.1128/AAC.00483-19 31427293 PMC6811410

[B26] García-Alcalde F. Okonechnikov K. Carbonell J. Cruz L. M. Götz S. Tarazona S. (2012). Qualimap: evaluating next-generation sequencing alignment data. *Bioinformatics* 28 2678–2679. 10.1093/bioinformatics/bts503 22914218

[B27] Garneau-Tsodikova S. Labby K. J. (2016). Mechanisms of resistance to aminoglycoside antibiotics: overview and perspectives. *Med. Chem. Commun.* 7 11–27. 10.1039/C5MD00344J 26877861 PMC4752126

[B28] Gieraltowski L. Higa J. Peralta V. Green A. Schwensohn C. Rosen H. (2016). National outbreak of multidrug resistant *Salmonella* Heidelberg infections linked to a single poultry company. *PLoS One* 11:e0162369. 10.1371/journal.pone.0162369 27631492 PMC5025200

[B29] Government of Canada (2013). *Canadian Integrated Program for Antimicrobial Resistance Surveillance (CIPARS) 2013 Annual Report - Chapter 2. Antimicrobial Resistance.* Guelph: Public Health Agency of Canada.

[B30] Government of Canada [CFIA] (2016). *National Microbiological Baseline Study in Broiler Chicken December 2012 – December 2013.* Available online at: http://www.inspection.gc.ca/food/chemical-residues-microbiology/food-safety-testing-bulletins/2016-08-17/december-2012-december-2013/eng/1471358115567/1471358175297 (accessed February 7, 2019).

[B31] Government of Canada [PHAC] (2007). *Canadian Integrated Program for Antimicrobial Resistance Surveillance (CIPARS).* Ottawa: Public Health Agency of Canada.

[B32] Grimont P. Weill F. (2007). *Antigenic Formulae of the Salmonella Serovars (9th ed.), WHO Collaborating Center for Reference and Research on Salmonella*, 9th Edn Paris: Institute Pasteur.

[B33] Grüning B. Dale R. Sjödin A. Chapman B. A. Rowe J. Tomkins-Tinch C. H. (2018). Bioconda: sustainable and comprehensive software distribution for the life sciences. *Nat. Methods* 15 475–476. 10.1038/s41592-018-0046-7 29967506 PMC11070151

[B34] Gupta S. K. Padmanabhan B. R. Diene S. M. Lopez-Rojas R. Kempf M. Landraud L. (2014). ARG-ANNOT, a new bioinformatic tool to discover antibiotic resistance genes in bacterial genomes. *Antimicrob. Agents Chemother.* 58 212–220. 10.1128/AAC.01310-13 24145532 PMC3910750

[B35] Hakanen A. Kotilainen P. Jalava J. Siitonen A. Huovinen P. (1999). Detection of decreased fluoroquinolone susceptibility in *Salmonellas* and validation of nalidixic acid screening test. *J. Clin. Microbiol.* 37 3572–3577. 10.1128/jcm.37.11.3572-3577.1999 10523554 PMC85694

[B36] Holmberg S. D. Osterholm M. T. Senger K. A. Cohen M. L. (1984). Drug-resistant *Salmonella* from animals fed antimicrobials. *N. Engl. J. Med.* 311 617–622. 10.1056/NEJM198409063111001 6382001

[B37] Hong S. Rovira A. Davies P. Ahlstrom C. Muellner P. Rendahl A. (2016). Serotypes and antimicrobial resistance in *Salmonella enterica* recovered from clinical samples from cattle and swine in Minnesota, 2006 to 2015. *PLoS One* 11:e0168016. 10.1371/journal.pone.0168016 27936204 PMC5148076

[B38] Inouye M. Dashnow H. Raven L.-A. Schultz M. B. Pope B. J. Tomita T. (2014). SRST2: rapid genomic surveillance for public health and hospital microbiology labs. *Genome Med.* 6:90. 10.1186/s13073-014-0090-6 25422674 PMC4237778

[B39] Knowles M. Lambert D. Huszczynski G. Gauthier M. Blais B. W. (2015). PCR for the specific detection of an *Escherichia coli* O157:H7 laboratory control strain. *J. Food Prot.* 78 1738–1744. 10.4315/0362-028X.JFP-15-147 26319729

[B40] Knowles M. Stinson S. Lambert D. Carrillo C. Koziol A. Gauthier M. (2016). Genomic tools for customized recovery and detection of foodborne shiga toxigenic *Escherichia coli*. *J. Food Prot.* 79 2066–2077. 10.4315/0362-028X.JFP-16-220 28221970

[B41] Koskiniemi S. Pränting M. Gullberg E. Näsvall J. Andersson D. I. (2011). Activation of cryptic aminoglycoside resistance in *Salmonella enterica*. *Mol. Microbiol.* 80 1464–1478. 10.1111/j.1365-2958.2011.07657.x 21507083

[B42] Laxminarayan R. Duse A. Wattal C. Zaidi A. K. M. Wertheim H. F. L. Sumpradit N. (2013). Antibiotic resistance—the need for global solutions. *Lancet Infect. Dis.* 13 1057–1098. 10.1177/1073110518782916 24252483

[B43] Lázár V. Singh G. P. Spohn R. Nagy I. Horváth B. Hrtyan M. (2013). Bacterial evolution of antibiotic hypersensitivity. *Mol. Syst. Biol.* 9:700. 10.1038/msb.2013.57 24169403 PMC3817406

[B44] Li W. Li Y. Wu Y. Cui Y. Liu Y. Shi X. (2016). Phenotypic and genetic changes in the life cycle of small colony variants of *Salmonella enterica* serotype Typhimurium induced by streptomycin. *Ann. Clin. Microbiol. Antimicrob.* 15:37. 10.1186/s12941-016-0151-3 27245674 PMC4888536

[B45] Licker M. Anghel A. Moldovan R. Hogea E. Muntean D. Horhat F. (2015). Genotype-phenotype correlation in multiresistant *Escherichia coli* and *Klebsiella pneumoniae* strains isolated in Western Romania. *Eur. Rev. Med. Pharmacol. Sci.* 19 1888–1894. 26044236

[B46] Low A. J. Koziol A. G. Manninger P. A. Blais B. Carrillo C. D. (2019). ConFindr: rapid detection of intraspecies and cross-species contamination in bacterial whole-genome sequence data. *PeerJ* 7:e6995. 10.7717/peerj.6995 31183253 PMC6546082

[B47] McArthur A. G. Waglechner N. Nizam F. Yan A. Azad M. A. Baylay A. J. (2013). The comprehensive antibiotic resistance database. *Antimicrob. Agents Chemother.* 57 3348–3357. 10.1128/AAC.00419-13 23650175 PMC3697360

[B48] McDermott P. F. Tyson G. H. Kabera C. Chen Y. Li C. Folster J. P. (2016). Whole-genome sequencing for detecting antimicrobial resistance in nontyphoidal *Salmonella*. *Antimicrob. Agents Chemother.* 60 5515–5520. 10.1128/AAC.01030-16 27381390 PMC4997858

[B49] Mikheil D. M. Shippy D. C. Eakley N. M. Okwumabua O. E. Fadl A. A. (2012). Deletion of gene encoding methyltransferase (*gidB*) confers high-level antimicrobial resistance in *Salmonella*. *J. Antibiot.* 65 185–192. 10.1038/ja.2012.5 22318332

[B50] Muñoz N. Diaz-Osorio M. Moreno J. Sánchez-Jiménez M. Cardona-Castro N. (2010). Development and evaluation of a multiplex real-time polymerase chain reaction procedure to clinically type prevalent *Salmonella enterica* serovars. *J. Mol. Diagn.* 12 220–225. 10.2353/jmoldx.2010.090036 20110454 PMC2871729

[B51] Nishimura K. Johansen S. K. Inaoka T. Hosaka T. Tokuyama S. Tahara Y. (2007). Identification of the RsmG methyltransferase target as 16S rRNA nucleotide G527 and characterization of *Bacillus subtilis rsmG* mutants. *J. Bacteriol.* 189 6068–6073. 10.1128/jb.00558-07 17573471 PMC1952054

[B52] Okamoto S. Tamaru A. Nakajima C. Nishimura K. Tanaka Y. Tokuyama S. (2007). Loss of a conserved 7-methylguanosine modification in 16S rRNA confers low-level streptomycin resistance in bacteria. *Mol. Microbiol.* 63 1096–1106. 10.1111/j.1365-2958.2006.05585.x 17238915

[B53] Okonechnikov K. Conesa A. García-Alcalde F. (2016). Qualimap 2: advanced multi-sample quality control for high-throughput sequencing data. *Bioinformatics* 32 292–294. 10.1093/bioinformatics/btv566 26428292 PMC4708105

[B54] Paradise M. R. Cook G. Poole R. K. Rather P. N. (1998). Mutations in *aarE*, the *ubiA* homolog of *Providencia stuartii*, result in high-level aminoglycoside resistance and reduced expression of the chromosomal aminoglycoside 2’-*N*-acetyltransferase. *Antimicrob. Agents Chemother.* 42 959–962. 10.1128/aac.42.4.959 9559821 PMC105580

[B55] Petkau A. Mabon P. Sieffert C. Knox N. C. Cabral J. Iskander M. (2017). SNVPhyl: a single nucleotide variant phylogenomics pipeline for microbial genomic epidemiology. *Microb. Genom.* 3:e000116. 10.1099/mgen.0.000116 29026651 PMC5628696

[B56] Piddock L. J. Ricci V. McLaren I. Griggs D. J. (1998). Role of mutation in the *gyrA* and *parC* genes of nalidixic-acid-resistant *Salmonella* serotypes isolated from animals in the United Kingdom. *J. Antimicrob. Chemother.* 41 635–641. 10.1093/jac/41.6.635 9687102

[B57] Plumb I. D. (2019). Outbreak of *Salmonella* newport infections with decreased susceptibility to azithromycin linked to beef obtained in the united states and soft cheese obtained in Mexico — united states, 2018–2019. *MMWR Morb. Mortal. Wkly. Rep.* 68 713–717. 10.15585/mmwr.mm6833a1 31437141 PMC6705891

[B58] Pornsukarom S. van Vliet A. H. M. Thakur S. (2018). Whole genome sequencing analysis of multiple *Salmonella* serovars provides insights into phylogenetic relatedness, antimicrobial resistance, and virulence markers across humans, food animals and agriculture environmental sources. *BMC Genomics* 19:801. 10.1186/s12864-018-5137-4 30400810 PMC6218967

[B59] R Core Team (2014). *R: A Language and Environment for Statistical Computing.* Vienna: R Foundation for Statistical Computing.

[B60] Randall L. P. Cooles S. W. Osborn M. K. Piddock L. J. V. Woodward M. J. (2004). Antibiotic resistance genes, integrons and multiple antibiotic resistance in thirty-five serotypes of *Salmonella enterica* isolated from humans and animals in the UK. *J. Antimicrob. Chemother.* 53 208–216. 10.1093/jac/dkh070 14729766

[B61] Reche M. P. de los Ríos J. E. G. Jiménez P. A. Rojas A. M. Rotger R. (2002). *gyrA* mutations associated with nalidixic acid-resistant *Salmonellae* from wild birds. *Antimicrob. Agents Chemother.* 46 3108–3109. 10.1128/AAC.46.9.3108-3109.2002 12183286 PMC127419

[B62] Robertson J. Lin J. Wren-Hedgus A. Arya G. Carrillo C. Nash J. H. E. (2019). Development of a multi-locus typing scheme for an *Enterobacteriaceae* linear plasmid that mediates inter-species transfer of flagella. *PLoS One* 14:e0218638. 10.1371/journal.pone.0218638 31738764 PMC6860452

[B63] Robertson J. Nash J. H. E. (2018). MOB-suite: software tools for clustering, reconstruction and typing of plasmids from draft assemblies. *Microb. Genom.* 4:e000206. 10.1099/mgen.0.000206 30052170 PMC6159552

[B64] Robertson J. Yoshida C. Kruczkiewicz P. Nadon C. Nichani A. Taboada E. N. (2018). Comprehensive assessment of the quality of *Salmonella* whole genome sequence data available in public sequence databases using the *Salmonella in silico* typing resource (SISTR). *Microb. Genom.* 4:e000151. 10.1099/mgen.0.000151 29338812 PMC5857378

[B65] Rosengren L. B. Waldner C. L. Reid-Smith R. J. (2009). Associations between antimicrobial resistance phenotypes, antimicrobial resistance genes, and virulence genes of fecal *Escherichia coli* isolates from healthy grow-finish pigs. *Appl. Environ. Microbiol.* 75 1373–1380. 10.1128/AEM.01253-08 19139228 PMC2648170

[B66] Routh J. A. Pringle J. Mohr M. Bidol S. Arends K. Adams-Cameron M. (2015). Nationwide outbreak of multidrug-resistant *Salmonella* Heidelberg infections associated with ground turkey: united states, 2011. *Epidemiol. Infect.* 143 3227–3234. 10.1017/S0950268815000497 25865382 PMC9150975

[B67] Ruiz J. (2003). Mechanisms of resistance to quinolones: target alterations, decreased accumulation and DNA gyrase protection. *J. Antimicrob. Chemother.* 51 1109–1117. 10.1093/jac/dkg222 12697644

[B68] Ruiz J. Castro D. Goñi P. Santamaria J. A. Borrego J. J. Vila J. (1997). Analysis of the mechanism of quinolone resistance in nalidixic acid-resistant clinical isolates of *Salmonella* serotype Typhimurium. *J. Med. Microbiol.* 46 623–628. 10.1099/00222615-46-7-623 9236748

[B69] Schneider J. L. White P. L. Weiss J. Norton D. Lidgard J. Gould L. H. (2011). Multistate outbreak of multidrug-resistant *Salmonella* newport infections associated with ground beef, October to December 2007. *J. Food Prot.* 74 1315–1319. 10.4315/0362-028X.JFP-11-046 21819658

[B70] Shipp C. R. Rowe B. (1980). A mechanised microtechnique for *Salmonella* serotyping. *J. Clin. Pathol.* 33 595–597. 10.1136/jcp.33.6.595 7400362 PMC1146150

[B71] Soballe B. Poole R. K. (1999). Microbial ubiquinones: multiple roles in respiration, gene regulation and oxidative stress management. *Microbiology* 145 1817–1830. 10.1099/13500872-145-8-1817 10463148

[B72] Souvorov A. Agarwala R. Lipman D. J. (2018). SKESA: strategic k-mer extension for scrupulous assemblies. *Genome Biol.* 19:153. 10.1186/s13059-018-1540-z 30286803 PMC6172800

[B73] Springer B. Kidan Y. G. Prammananan T. Ellrott K. Böttger E. C. Sander P. (2001). Mechanisms of streptomycin resistance: selection of mutations in the 16S rRNA gene conferring resistance. *Antimicrob. Agents Chemother.* 45 2877–2884. 10.1128/AAC.45.10.2877-2884.2001 11557484 PMC90746

[B74] Su L.-H. Chiu C.-H. Chu C. Ou J. T. (2004). Antimicrobial resistance in nontyphoid *Salmonella* serotypes: a global challenge. *Clin. Infect. Dis.* 39 546–551. 10.1086/422726 15356819

[B75] Threlfall J. Levent B. Hopkins K. L. de Pinna E. Ward L. R. Brown D. J. (2005). Multidrug-resistant *Salmonella* java. *Emerg. Infect. Dis.* 11 170–171. 10.3201/eid1101.031092 15714662 PMC3294332

[B76] Tyson G. H. Li C. Ayers S. McDermott P. F. Zhao S. (2016). Using whole-genome sequencing to determine appropriate streptomycin epidemiological cutoffs for *Salmonella* and *Escherichia coli*. *FEMS Microbiol. Lett.* 363:fnw009. 10.1093/femsle/fnw009 26781915 PMC11555754

[B77] Tyson G. H. McDermott P. F. Li C. Chen Y. Tadesse D. A. Mukherjee S. (2015). WGS accurately predicts antimicrobial resistance in *Escherichia coli*. *J. Antimicrob. Chemother.* 70 2763–2769. 10.1093/jac/dkv186 26142410 PMC11606221

[B78] Uelze L. Borowiak M. Deneke C. Szabó I. Fischer J. Tausch S. H. (2020). Performance and accuracy of four open-source tools for in silico serotyping of *Salmonella* spp. based on whole-genome short-read sequencing data. *Appl. Environ. Microbiol.* 86:e02265-19. 10.1128/AEM.02265-19 31862714 PMC7028957

[B79] van Hoek A. H. A. M. Mevius D. Guerra B. Mullany P. Roberts A. P. Aarts H. J. M. (2011). Acquired antibiotic resistance genes: an overview. *Front. Microbiol.* 2:203 10.3389/fmicb.2011.00203 PMC3202223 22046172

[B80] van Pelt W. van der Zee H. Wannet W. J. B. van de Giessen A. W. Mevius D. J. Bolder N. M. (2003). Explosive increase of *Salmonella* Java in poultry in the Netherlands: consequences for public health. *Euro Surveill.* 8 31–35. 10.2807/esm.08.02.00398-en 12631972

[B81] Walker B. J. Abeel T. Shea T. Priest M. Abouelliel A. Sakthikumar S. (2014). Pilon: an integrated tool for comprehensive microbial variant detection and genome assembly improvement. *PLoS One* 9:e112963. 10.1371/journal.pone.0112963 25409509 PMC4237348

[B82] WHO (2017). *Antimicrobial Resistance.* Geneva: WHO.

[B83] Wiegand I. Hilpert K. Hancock R. E. W. (2008). Agar and broth dilution methods to determine the minimal inhibitory concentration (MIC) of antimicrobial substances. *Nat. Protoc.* 3 163–175. 10.1038/nprot.2007.521 18274517

[B84] Wong S. Y. Lee J. S. Kwak H. K. Via L. E. Boshoff H. I. M. Barry C. E. (2011). Mutations in *gidB* confer low-level streptomycin resistance in *Mycobacterium tuberculosis*. *Antimicrob. Agents Chemother.* 55 2515–2522. 10.1128/AAC.01814-10 21444711 PMC3101441

[B85] Yachison C. A. Yoshida C. Robertson J. Nash J. H. E. Kruczkiewicz P. Taboada E. N. (2017). The validation and implications of using whole genome sequencing as a replacement for traditional serotyping for a national *Salmonella* reference laboratory. *Front. Microbiol.* 8:1044. 10.3389/fmicb.2017.01044 28649236 PMC5465390

[B86] Yoon K.-B. Song B.-J. Shin M.-Y. Lim H.-C. Yoon Y.-H. Jeon D.-Y. (2017). Antibiotic resistance patterns and serotypes of *Salmonella* spp. isolated at Jeollanam-do in Korea. *Osong Public Health Res. Perspect.* 8 211–219. 10.24171/j.phrp.2017.8.3.08 28781944 PMC5525558

[B87] Yoshida C. E. Kruczkiewicz P. Laing C. R. Lingohr E. J. Gannon V. P. J. Nash J. H. E. (2016). The *Salmonella* in silico typing resource (SISTR): an open web-accessible tool for rapidly typing and subtyping draft *Salmonella* genome assemblies. *PLoS One* 11:e0147101. 10.1371/journal.pone.0147101 26800248 PMC4723315

[B88] Zankari E. (2014). Comparison of the web tools ARG-ANNOT and ResFinder for detection of resistance genes in bacteria. *Antimicrob. Agents Chemother.* 58 4986–4986. 10.1128/aac.02620-14 25028728 PMC4136053

[B89] Zankari E. Allesøe R. Joensen K. G. Cavaco L. M. Lund O. Aarestrup F. M. (2017). PointFinder: a novel web tool for WGS-based detection of antimicrobial resistance associated with chromosomal point mutations in bacterial pathogens. *J. Antimicrob. Chemother.* 72 2764–2768. 10.1093/jac/dkx217 29091202 PMC5890747

[B90] Zankari E. Hasman H. Cosentino S. Vestergaard M. Rasmussen S. Lund O. (2012). Identification of acquired antimicrobial resistance genes. *J. Antimicrob. Chemother.* 67 2640–2644. 10.1093/jac/dks261 22782487 PMC3468078

[B91] Zhang S. den Bakker H. C. Li S. Chen J. Dinsmore B. A. Lane C. (2019). SeqSero2: rapid and improved *Salmonella* serotype determination using whole-genome sequencing data. *Appl. Environ. Microbiol.* 85:e01746-19. 10.1128/AEM.01746-19 31540993 PMC6856333

[B92] Zhang S. Yin Y. Jones M. B. Zhang Z. Kaiser B. L. D. Dinsmore B. A. (2015). *Salmonella* serotype determination utilizing high-throughput genome sequencing data. *J. Clin. Microbiol.* 53 1685–1692. 10.1128/JCM.00323-15 25762776 PMC4400759

[B93] Zhao S. McDermott P. F. White D. G. Qaiyumi S. Friedman S. L. Abbott J. W. (2007). Characterization of multidrug resistant *Salmonella* recovered from diseased animals. *Vet. Microbiol.* 123 122–132. 10.1016/j.vetmic.2007.03.001 17400409

[B94] Zhao S. Tyson G. H. Chen Y. Li C. Mukherjee S. Young S. (2016). Whole-genome sequencing analysis accurately predicts antimicrobial resistance phenotypes in *Campylobacter* spp. *Appl. Environ. Microbiol.* 82 459–466. 10.1128/AEM.02873-15 26519386 PMC4711122

